# GnRH-mediated suppression of S100A4 expression inhibits endometrial epithelial cell proliferation in sheep via GNAI2/MAPK signaling

**DOI:** 10.3389/fvets.2024.1410371

**Published:** 2024-05-30

**Authors:** Xiyao Jiao, Zhili Chu, Meng Li, Jiurong Wang, Zilong Ren, Leyang Wang, Chengcheng Lu, Xiangyun Li, Feng Ren, Xinglong Wu

**Affiliations:** ^1^College of Animal Science and Technology, Hebei Technology Innovation Center of Cattle and Sheep Embryo, Hebei Agricultural University, Baoding, China; ^2^School of Basic Medical Sciences, Xinxiang Medical University, Xinxiang, China; ^3^Henan International Joint Laboratory of Immunity and Targeted Therapy for Liver-Intestinal Tumors, Xinxiang Medical University, Xinxiang, China

**Keywords:** sheep endometrial epithelial cells, GnRH, cell proliferation, S100A4, GNAI2, MAPK signaling pathway

## Abstract

**Background:**

Gonadotrophin-releasing hormone (GnRH) administration significantly decreases the pregnancy rate of recipient ewes after embryo transfer, possibly because GnRH affects endometrial epithelial cell function. Therefore, this study investigated the effect of GnRH on endometrial epithelial cells.

**Methods:**

Transcriptome sequencing was used to determine the regulatory effect of GnRH on the ewe endometrium, and the S100A4 gene, which showed altered transcription, was screened as a candidate regulator of this effect. Endometrial epithelial cells were further isolated, the S100A4 protein was immunoprecipitated, and host proteins that interacted with S100A4 were identified by mass spectrometry. We further verified the effects of S100A4 and GNAI2 on the proliferation of endometrial epithelial cells via overexpression/knockdown experiments and subsequent CCK-8 and EdU assays. The effect of S100A4 deletion in endometrial cells on reproduction was verified in mice with S100A4 knockout.

**Results:**

Our results showed that S100A4 gene transcription in endometrial cells was significantly inhibited after GnRH administration. GNAI2 was identified as a downstream interacting protein of S100A4, and S100A4 was confirmed to activate the MAPK signaling pathway to promote cell proliferation by targeting GNAI2.

**Conclusion:**

GnRH can suppress the expression of S100A4 in the endometrium, consequently inhibiting the proliferation of endometrial cells through the S100A4/GNAI2/MAPK signaling pathway. These findings suggest a potential explanation for the limited efficacy of GnRH in promoting embryo implantation.

## 1 Background

The application of assisted reproductive technology is important for increasing the pregnancy rate of sheep. Further research into the effects of different hormones on reproduction is therefore warranted. Gonadotrophin-releasing hormone (GnRH) plays a pivotal role in hormonal dynamics and reproductive efficiency in mammals. GnRH is a decapeptide hormone that is synthesized and secreted by specific hypothalamic neurons. This hormone is released into the pituitary portal system in a pulsed manner, binds to the GnRH receptor on the surface of the pituitary gland, regulates the hypothalamus-pituitary-gonadal (HPG) axis, stimulates the release of gonadotropins and steroid hormones, such as follicle stimulating hormone (FSH) and luteinizing hormone (LH) (1), and then regulates embryo implantation and placental formation, playing an essential role in maintaining the normal reproductive activities of animals (1–3). GnRH and GnRH receptors are found in the hypothalamus, pituitary glands, and various reproductive organ tissues, such as the ovaries, endometrium, and myometrium. GnRH mRNA is expressed in both epithelial and stromal cells of the human endometrium in a dynamic manner. In the early and middle secretory stages of the menstrual cycle, endometrial GnRH mRNA levels increase significantly, suggesting that GnRH may be involved in the embryo implantation process (4). GnRH receptors are expressed in human endometrial decidual stromal cells, and GnRH agonists can promote the release of enzymes that remodel the extracellular matrix and change cell movement (1, 5). GnRH agonists can inhibit the proliferation, migration, and differentiation of endometrial stem cells through the PI3K/Akt signaling pathway (6) and can also affect the receptivity of the endometrium by affecting the immune response and energy metabolism (7). GnRH and GnRH receptors are also expressed in the myometrium. GnRH agonists can inhibit DNA synthesis, TGF-β production in myometrial smooth muscle cells and proliferation of uterine fibroids (4, 8). In summary, GnRH participates in the embryo implantation process by mediating various functions of endometrial cells, and exogenous GnRH can affect uterine function. Therefore, GnRH is often used clinically to induce ovulation during superovulation (9–11) and to synchronize estrus (12, 13).

GnRH is widely used to increase pregnancy rates in sheep. GnRH agonists/antagonists were first used to control follicle development (9–11, 14) and to assess the efficacy of estrus induction during different periods (15). This molecule can also be used to induce estrus synchronization (12, 13, 16). However, we previously found that using GnRH in sheep fixed-time artificial insemination and embryo transfer programs does not increase the embryo and embryo transfer programs decreased the embryo implantation rate (17). Unfortunately, the mechanism of its negative effect is still unclear.

Physiological changes in endometrial cells are essential for cyclic changes in the uterus. In particular, the endometrium must respond to hormonal changes and proliferate to a state suitable for embryo implantation. Generally, endometrial cell proliferation is regulated by hormones such as estrogen, which acts as a transcription factor that directs target gene expression and exerts its effects through interactions with the membrane-bound estrogen receptor (ER). The ER utilizes intracellular signaling systems [such as mitogen-activated protein kinase (MAPK)/ERK, phosphoinositide 3-kinase (PI3K), and RAS signaling pathways] to participate in the regulation of gene expression (18). However, the molecular mechanism by which hormones activate endometrial cell proliferation after they act on endometrial cells is not fully understood. Studies have not shown whether GnRH treatment can affect endometrial cell proliferation.

The S100 protein family consists of 25 calcium-binding proteins with high sequence and structural similarities. S100A4 is a critical member expressed in various cell types, including lymphoid and myeloid cells such as macrophages, neutrophils, mast cells, and memory T cells. This molecule plays a crucial role in promoting cancer occurrence and metastasis. The S100A4 protein also regulates obesity (19), cell proliferation (20), migration (21, 22), inflammation, and cell death (23). Additionally, S100A4 is a key dynamic regulator of embryo implantation (24). In cases of inflammatory reactions in the bovine uterus, the expression level of S100A4 significantly decreases, suggesting its relevance to the pathogenesis of endometrial inflammation (25).

In the pathogenesis of uterine sarcoma, S100A4 also plays a crucial role in affecting epithelial–mesenchymal transition (26). Several studies have shown that S100A4 can promote the migration and metastasis of endometrial cancer cells (27). These findings collectively suggest that S100A4 plays an important regulatory role in uterine diseases.

However, the role of S100A4 in regulating endometrial cell proliferation remains to be explored. Transcriptome sequencing revealed that changes in S100A4 expression affect endometrial proliferation. Furthermore, the downstream interacting protein GNAI2 was identified through coimmunoprecipitation. These findings were validated through *in vitro* and *in vivo* experiments in mice with uterine-specific S100A4 knockout, confirming that S100A4 can regulate the MAPK signaling pathway to promote endometrial cell proliferation.

## 2 Materials and methods

### 2.1 Animal experiments

This experiment was conducted from November to December 2019 at a Kazakh sheep breeding farm in Liaoning Province, China (41°06′N 122°18′E). The 20 selected sheep had no reproductive disorders. The weight was 40–60 kg. The ewes selected for experiments were treated with an intravaginal vaginal sponge soaked with 45 mg of progesterone (Muqimuye Sci-Tech Co., Ltd., Shanghai, China) for 12 days. When the sponge was removed, 330 IU PMSG (Sansheng Biological Technology Co., Ltd., Ningbo, China) was injected intramuscularly. Ewes induced to undergo estrus were subjected to insemination 50 h after the sponge was removed. Laparoscopic uterine horn insemination was performed using an insemination device (Zhengmu Bio-Tech Co., Ltd., Baoding, China). Semen in 0.25 mL aliquots was thawed via conventional methods. The spermatozoa cell concentration was approximately 400 × 10^6^ cells/mL. The experimental ewes were divided into two groups. The experimental group was administered 17 μg of A3 (a GnRH agonist triptorelin; Sansheng Biological Technology Co., Ltd., Ningbo, China) by intramuscular injection 12 h before insemination, and the control group was administered 1 mL of sterile physiological saline solution via the same route. The endometrium was assessed 5 days after insemination. The Ethical Committee of Xinxiang Medical University approved this study.

### 2.2 Isolation and culture of sheep endometrial epithelial cells

Sheep uteri were collected at the slaughterhouse and immersed in physiological saline containing penicillin (100 IU/mL) and streptomycin (1 mg/mL). The uteri were then transported in an ice box to the laboratory. In a biosafety cabinet, the uteri were rinsed three times with PBS. During surgery, the uteri were cut into pieces, and efforts were made to remove nonendometrial tissue as thoroughly as possible. After the tissue was cut, the tissue homogenate was added to a cell culture dish, allowed to dry and fixed for 1 h. Cell culture medium containing 10% fetal bovine serum, penicillin, and streptomycin was added to the culture dish. The cells typically began to expand after approximately 7 days. When the cells reached approximately 70% confluence, they were harvested, digested, and frozen for future use. The Ethical Committee of Xinxiang Medical University approved this study.

### 2.3 Cell transfection with siRNA and plasmids

Plasmids for synthesizing siRNAs and overexpressing sheep S100A4 and GNAI2 were used. The target sequences are listed in Table 1. The cells were transferred to a 6-well cell culture plate one day in advance, with approximately 5 × 10^6^ cells per well. During transfection, Lipofectamine 2000 was mixed with serum-free basic DMEM to create solution A, while plasmid/siRNA was mixed with serum-free high-glucose basic DMEM to create solution B. After 5 min, solutions A and B were combined, and the mixture was allowed to incubate at room temperature for 12 min. This mixture was subsequently added to the cell culture dish, and the culture medium was replaced with fresh medium after 6 h. Subsequent experiments were conducted 42 h after transfection.

**Table 1:** Primer sequences.

	Forward sequence	Reverse sequence
S100A4	TGAGCAACTTGGACAGCAACA	TTCCGGGGTTCCTTATCTGGG
GNAI2	GGTCGTCTACAGCAACACCA	GTCTTCACTCGAGTCCGCAG
C-fos	CCAGCCCTGACCTACAATGG	CTGCCTCCCGTCATGGTTT
c-JUN	CGACCTTCTACGACGATGCC	GGGGTTACTGTAGCCGTAGG
GAPDH	AGGTCGGTGTGAACGGATTTG	TGTAGACCATGTAGTTGAGGTCA
si-S100A4	GCAUCGCCAUGAUGUGCAATT	UUGCACAUCAUGGCGAUGCTT
si-NC	UUCUCCGAACGUGUCACGUTT	ACGUGACACGUUCGGAGAATT
si-GNAI2	GCACGAGAGCAUGAAGCUGUUTT	AACAGCUUCAUGCUCUCGUGCTT
Alb-cre-Primer 1	GAAGCAGAAGCTTAGGAAGATGG	TTGGCCCCTTACCATAACTG
Alb-cre-Primer 2	GGACAACTTATCCTTATCACAAGGG	TTGGCCCCTTACCATAACTG
koS100A4-LoxP	ACAATGAAGTTGACTTCCAGGAGTA	GCAAACTACACCCCAACACTTC

### 2.4 Transcriptome sequencing

For processing of sheep tissue samples, the samples were ground and then lysed with TRIzol. For cell samples, the cell supernatant was discarded, and the cells were washed once with PBS and then lysed with TRIzol. RNA was subsequently extracted via the addition of chloroform and TRIzol at a 10% v/v ratio, followed by shaking, mixing, and centrifugation at 12,000 rpm for 10 min. The supernatant was transferred to a new centrifuge tube, and an equal volume of isopropanol was added. After inversion, mixing, and a 30-min incubation step, the mixture was centrifuged at 12,000 rpm for 10 min. The supernatant was gently discarded, and 1 mL of precooled 75% alcohol was added. The samples were centrifuged again at 12,000 rpm for 10 min, the supernatant was removed, and the pellet was allowed to dry in a fume hood. The RNA was subsequently dissolved in ddH2O for library construction.

Sheep uterine tissue sequencing and data analysis were performed by Beijing Geekgene Technology Co., Ltd. Sequencing following S100A4 knockdown was conducted by Suzhou GenePharma Co., Ltd. Transcriptome sequencing and analysis of GNAI2-overexpressing cells were performed by Beijing Novo Biotechnology Co., Ltd.

### 2.5 CCK-8 assays

Cells were seeded into a 96-well plate. After the corresponding treatment (as described in Materials and Methods 2), CCK-8 (C0005, Targetmol, United States) reagent was mixed with culture medium at a ratio of 1:9 in a centrifuge tube, and then, 100 μL of culture medium containing CCK-8 reagent was added to each well. The plate was placed in a cell culture incubator for further incubation. After 30 min, the OD450 was measured using a FlexStation 3 instrument.

### 2.6 EdU staining

After the cells were processed, EdU was dissolved in culture medium (10 μM), mixed evenly, and placed in a cell culture incubator for 30 min. Then, the culture medium in the original cell culture dish was discarded, and the above mixture was added. The cells were incubated for 1–3 h. Afterward, the culture medium containing EdU was discarded, and the cells were washed twice with PBS, fixed with 4% paraformaldehyde for 30 min, washed twice with PBS, and treated with a 0.3% Triton 100 solution for 15 min. The cells were stained according to the instructions of the working solution of the EdU kit and incubated for 30 min. After the staining solution was discarded, the cells were rinsed twice with PBS, PBS containing Hoechst 33342 was added, and images were captured using a fluorescence microscope (Ts2R-FL, Nikon, Japan).

### 2.7 Immunofluorescence and immunohistochemical staining

For immunofluorescence staining, tissue sections were subjected to conventional dewaxing, repaired with citric acid solution, and then washed with tap water to remove citric acid. Subsequently, the sections were blocked with 1% BSA for 30 min, and GnRHR (diluted with 1% BSA; 1:300; 19,950-1-AP; Proteintech, China) primary antibody or S100A4 primary antibody (1:100; sc-377059; Santa Cruz, United States) was added and incubated overnight at 4°C. After the primary antibody was removed by washing the next day, a fluorescent secondary antibody (1:200, AS037, Abclone, China) was applied, and the samples were incubated in the dark for 2 h. After the secondary antibodies were removed, the slides were covered with glycerol containing Hoechst 33342, and images were captured using a fluorescence microscope. For immunohistochemical staining of tissue sections, antigen retrieval solution was used to restore epitope−antibody binding. Endogenous peroxidase activity was blocked via the addition of a 3% hydrogen peroxide solution and incubation in the dark for 30 min at room temperature. Then, the tissue was blocked with 3% BSA for 30 min. The antibody was dissolved in PBS and placed in a humidified box overnight for 4 days. The next day, an HRP-labeled secondary antibody was added to the corresponding mixture, which was incubated at room temperature for 1 h. Subsequently, DAB chromogenic solution was used for color development, followed by hematoxylin counterstaining for 3 min and rinsing with tap water. The slides were dehydrated, mounted, and photographed using a microscope.

### 2.8 Mass spectrometry and coimmunoprecipitation (Co-IP)

For mass spectrometry, S100A4 (with an HA tag) was first overexpressed in endometrial epithelial cells. After 48 h, the cells were lysed with IP lysis buffer (Beyotime). Then, the cell lysates were incubated with precoated anti-HA primary antibody (CST) and Protein A/G. The cells were incubated for 12 h, followed by six rinses with NP-40 solution. Finally, the solution was discarded, and protein A/G beads were isolated for mass spectrometry analysis. Mass spectrometry-based detection and analysis were conducted by Jin Kairui Biotechnology, Ltd.

For Co-IP, 293 T cells were prepared and simultaneously transfected with the S100A4 and GNAI2 plasmids. After 48 h, the proteins were collected, and a portion of the total protein sample was stored for later use. The same procedures were followed as described above. After the proteins bound to the protein A/G beads were obtained, 50 μL of 1× loading buffer was added. The samples were heated in a 100°C metal bath for 5 min, after which electrophoresis was performed at 100 V constant voltage. A 300 mA constant current was used for 90 min for transfer. The PVDF membrane was blocked with 10% skim milk powder for 30 min, the corresponding primary antibody was added, and the membrane was incubated overnight at 4°C. The cells were rinsed with TBST for 3 × 10 min, incubated with the secondary antibody at room temperature for 2 h, and then rinsed with TBST for 3 × 10 min. Chemiluminescence solution (Bio-Rad, United States) was added, and images were captured.

### 2.9 Primer sequences and qPCR analysis

Total cellular RNA was extracted as described in (3). The RNA was reverse transcribed into cDNA using a reverse transcription kit (D7168M, Beyotime Biotechnology Co., Ltd.) and analyzed using a qPCR kit (A313-05, Beijing Kangrun Chengye Biotechnology Co., Ltd.). The components included 2 × qPCR Mix (10 μL), 1 μL each of Primer F and Primer R, 0.5 μL of cDNA, and 7.5 μL of H2O. The qPCR program involved denaturation at 94°C for 30 s, annealing at 60°C for 30 s, and replication at 72°C for 30 s; this process was repeated for 40 cycles. The primer sequences are listed in Table 1.

### 2.10 HE staining

The paraffin sections were dewaxed in water, stained with hematoxylin for 3–5 min, and then rinsed with tap water. The sections were counterstained with a bluing solution and then rinsed with running water. The sections were dehydrated in 85 and 95% gradient alcohol for 5 min each and subsequently immersed in an eosin staining solution for 5 min. Finally, the slices were placed in absolute ethanol solutions I, II, and III for 5 min each. The slides were cleared in xylene twice for 5 min each, sealed with neutral gum, allowed to dry, and imaged.

### 2.11 Animal breeding and analysis

Transgenic animals were purchased from Saiye Biotech and kept in specific pathogen-free (SPF) animal rooms with a 12 h/12 h dark/light cycle, after which the mice were allowed access to drinking water and food. A Mouse Direct PCR Kit (for genotyping) (B40013, Selleck) was used for mouse genotyping. The sequences of the primers used for genotype identification are shown in Table 1.

### 2.12 Statistical analysis

Statistical analysis was performed with GraphPad Prism 5 software (GraphPad Software, Inc., CA, United States). All values are expressed as the means ± SDs of three independent experiments. Student’s t test and one-way ANOVA were used to evaluate the significance of differences; *p* < 0.05 was considered to indicate statistical significance (28).

## 3 Results

### 3.1 S100A4 expression in the sheep endometria decreased in response to GnRH treatment before insemination

**Figure 1:**
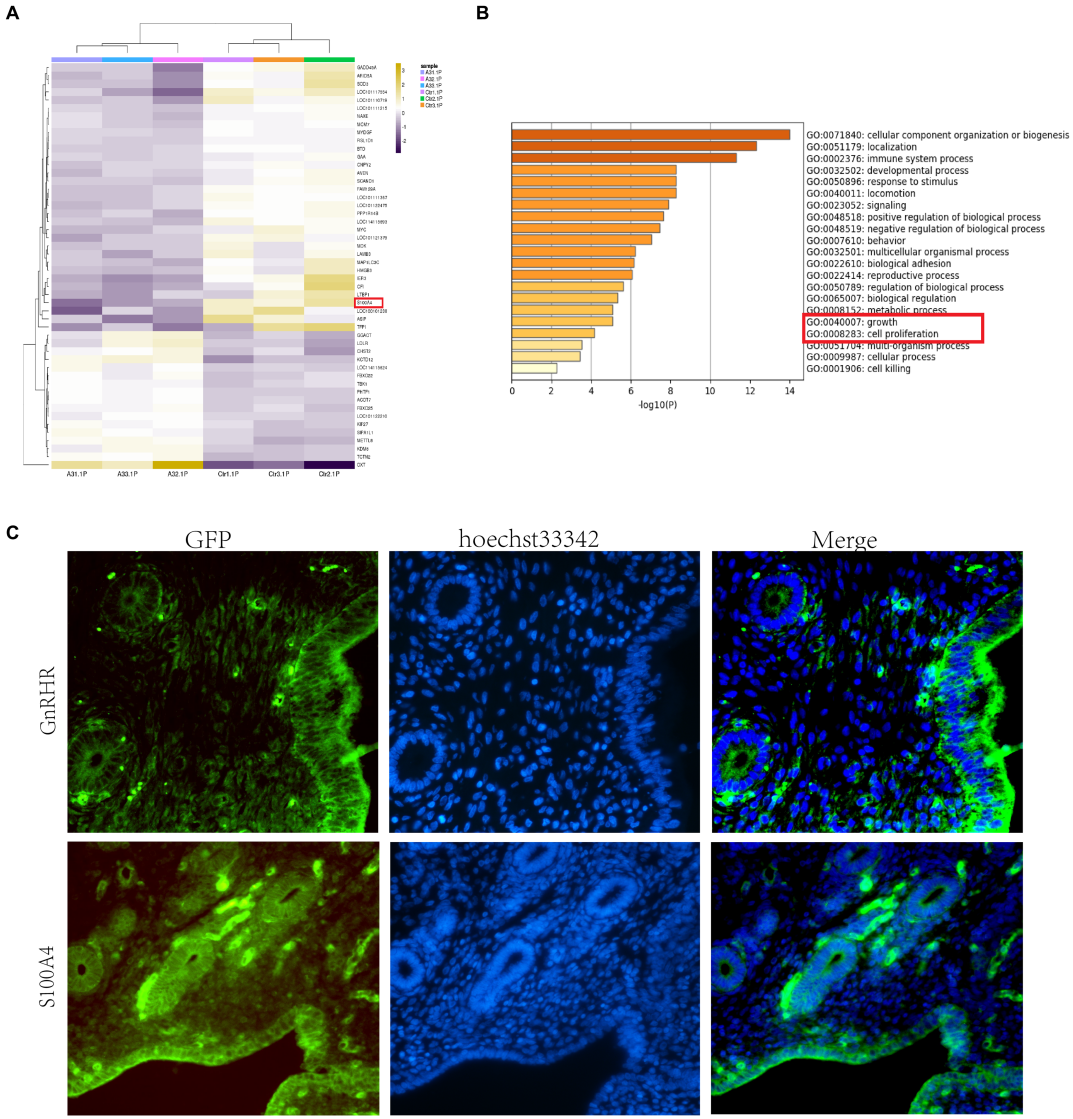
GnRH reduces the expression of S100A4 in the sheep endometrium. **(A)** Heatmap showing significant variation in the expression level of S100A4. **(B)** Transcriptome sequencing and GO analysis revealed that GnRH affects cell proliferation-related signaling pathways, such as “growth” and “cell proliferation,” in treated sheep. **(C)** Expression of GnRHR and S100A4 in the sheep endometrium; the results show that the localization patterns of S100A4 and GnRHR are similar.

We treated sheep with GnRH before insemination, but no satisfactory results were achieved. To investigate the reasons underlying embryo implantation failure in sheep treated with GnRH before insemination and explore the effects of GnRH on the endometrium, we divided the sheep into two groups: one group received 17 μg A3 (GnRH) before insemination, while the other group received 1 mL of sterile physiological saline solution as a control. Five days after insemination, we collected uterine tissue, scraped the endometrial tissue, and performed transcriptome sequencing. Analysis of the differentially expressed genes (DEGs) between samples from the control and treated sheep revealed substantial differences in the expression of S100A4 between the two groups of sheep (Figure 1A). S100A4 is known to regulate cell proliferation. Kyoto Encyclopedia of Genes and Genomes (KEGG) enrichment analysis revealed that GnRH affected the enrichment of cell proliferation-related signaling pathways, such as “growth” and “cell proliferation” (Figure 1B). We initially performed immunofluorescence analysis to assess the expression of the GnRH receptor protein (GnRHR) in the endometrium. The results revealed robust expression of the GnRH receptor protein in the endometrium, as indicated by green fluorescence (Figure 1C). To investigate the localization of the S100A4 protein in the sheep endometrium, we performed immunofluorescence staining. We found that S100A4 was expressed in the endometrium in a pattern similar to that of GnRHR (Figure 1C). These findings suggest that S100A4 may play a role in the effects of GnRH treatment on the endometrium, although the specific mechanism involved remains unclear.

### 3.2 S100A4 promotes sheep endometrial cell proliferation

To assess the effect of S100A4 expression on the sheep endometrium, we isolated endometrial epithelial cells and synthesized an siRNA targeting sheep S100A4 and a eukaryotic expression vector to overexpress sheep S100A4.

**Figure 2:**
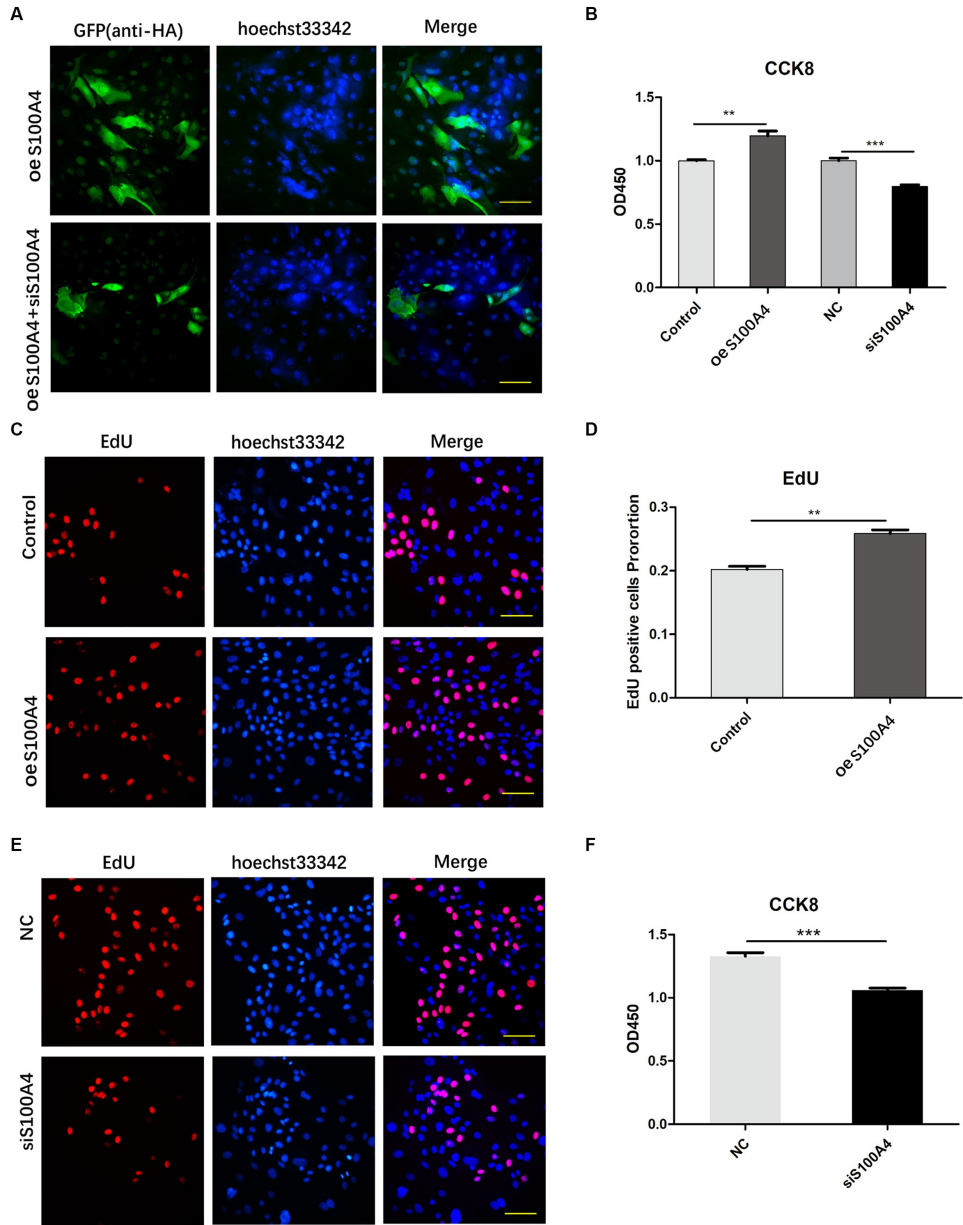
S100A4 overexpression promotes endometrial cell proliferation. **(A)** Confirmation of S100A4 overexpression and knockdown in endometrial epithelial cells through immunofluorescence. **(B)** CCK-8 analysis of the effects of S100A4 knockdown and overexpression on cell metabolism. **(C,D)** EdU staining-based determination of the effects of S100A4 overexpression on the proliferation of endometrial epithelial cells. **(E,F)** EdU staining-based determination of the effects of S100A4 knockdown on the proliferation of endometrial epithelial cells.

S100A4 was overexpressed in sheep cells, and one group of these cells was transfected with siRNA targeting S100A4 (siS100A4); then, the expression of HA-S100A4 was assessed through immunofluorescence. The results indicated that the anti-HA antibody could be used to identify HA-positive S100A4-overexpressing cells, and the fluorescence ratio decreased in the siS100A4-transfected group (Figure 2A). These findings suggested that both overexpression and knockdown of S100A4 can be achieved in endometrial epithelial cells.

Subsequently, we introduced siRNA into the cells, with the NC group serving as the control group. We then used a CCK-8 assay to assess changes in cell viability. The results revealed decreased cell viability after S100A4 knockdown (Figure 2B). Conversely, the S100A4-overexpressing group had a greater percentage of viable cells than did the empty vector group (Figure 2B).

To assess cell proliferation, we labeled endometrial epithelial cells with EdU for 2 h. A significantly greater proportion of cells with red fluorescence was found in the S100A4-overexpressing group than in the control group (Figures 2C,D). Conversely, the proportion of cells with red fluorescence decreased among the cells with S100A4 knockdown (Figures 2E,F). These findings suggest that S100A4 can indeed promote the proliferation of endometrial cells.

### 3.3 S100A4 knockdown affects cell proliferation-related signaling pathways in endometrial epithelial cells

**Figure 3:**
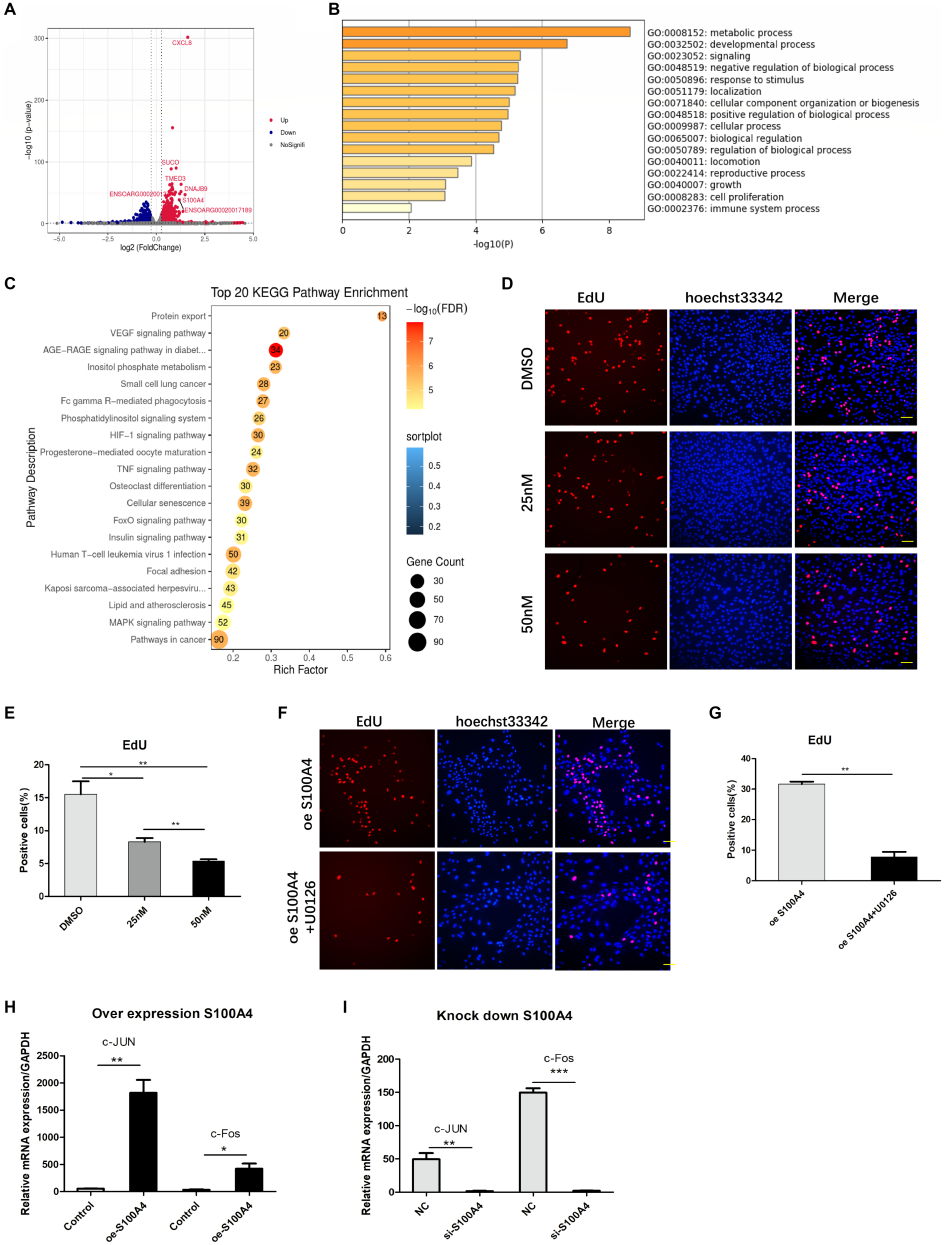
S100A4 regulates cell proliferation through the MAPK signaling pathway. **(A)** Volcano plot illustrating the impact of S100A4 knockdown on gene transcription in endometrial epithelial cells. **(B)** GO analysis indicating the effect of S100A4 knockdown on the expression of genes related to “growth” and “cell proliferation” in endometrial epithelial cells. **(C)** KEGG enrichment analysis revealing the enrichment of genes associated with the MAPK signaling pathway following S100A4 knockdown. **(D,E)** EdU staining-based analysis of endometrial epithelial cell proliferation after treatment with the MAPK inhibitor U0126. **(F,G)** EdU staining-based analysis of the effect of blocking the MAPK signaling pathway on cell proliferation after S100A4 overexpression. **(H)** qPCR analysis of the expression of the MAPK signaling pathway downstream genes C-Jun and c-Fos following S100A4 overexpression. **(I)** qPCR analysis of the expression of the MAPK signaling pathway downstream genes C-Jun and c-Fos following S100A4 knockdown.

To elucidate the mechanism by which S100A4 regulates endometrial cell proliferation, we conducted transcriptome sequencing on the NC and S100A4 knockdown groups. The volcano plot results showed that after S100A4 was knocked down, several genes, including S100A4 itself, were downregulated (Figure 3A). GO cluster analysis of these genes revealed that many of the DEGs were enriched in gene sets related to the regulation of “growth” and “cell proliferation” (Figure 3B). Furthermore, KEGG pathway enrichment analysis revealed that 52 of the DEGs were enriched in the MAPK signaling pathway (Figure 3C). These findings suggest that S100A4 may regulate cell proliferation through the MAPK signaling pathway.

To assess the effect of the MAPK signaling pathway on endometrial epithelial cell proliferation, we treated cells with the MAPK inhibitor U0126, which targets the key node protein ERK1/2. Endometrial epithelial cells were treated with various concentrations of the inhibitor for 12 h, followed by EdU labeling. Fluorescence staining revealed a significant reduction in the percentage of EdU-positive endometrial epithelial cells after MAPK pathway inhibition (Figures 3D,E), indicating that the MAPK signaling pathway indeed regulates the proliferation of these cells.

To determine whether S100A4 regulates cell proliferation via the MAPK signaling pathway, we added U0126 to cells overexpressing S100A4. EdU staining showed that, compared to that in the DMSO solvent group, the percentage of EdU-positive cells was significantly lower in the U0126 (50 nM) treatment group (Figures 3F,G). C-Jun and c-Fos are downstream target molecules of the MAPK signaling pathway and are known to be involved in regulating cell proliferation. qPCR analysis demonstrated that both the overexpression (Figure 3H) and knockdown (Figure 3I) of S100A4 affected the expression of C-Jun and c-Fos, and the expression of S100A4 was positively correlated with the expression of these two genes. These findings suggest that S100A4 may modulate sheep endometrial cell proliferation by regulating MAPK.

### 3.4 S100A4 promotes cell proliferation by interacting with the GNAI2 protein

**Figure 4:**
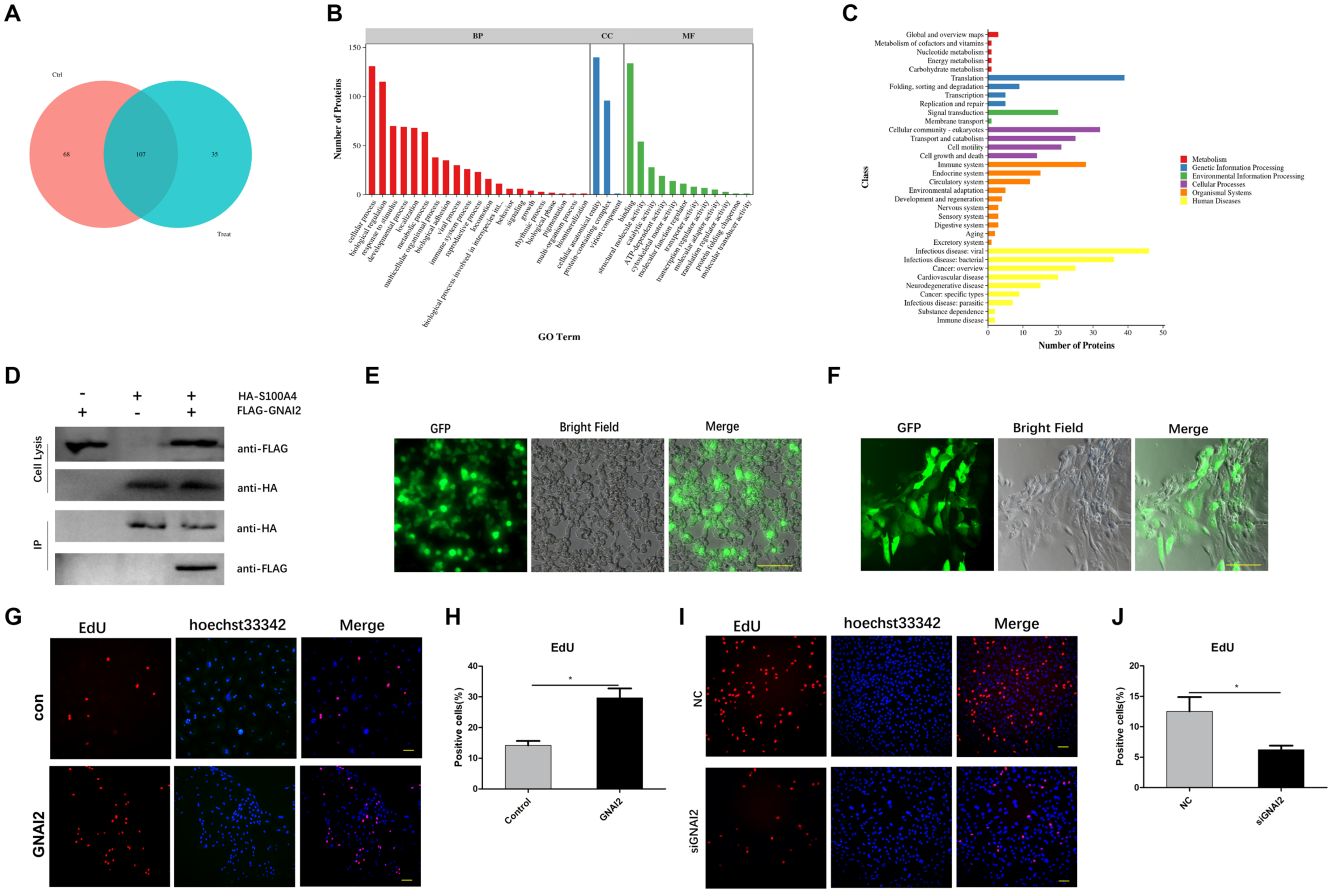
GNAI2 and S100A4 interact and promote cell proliferation. **(A)** Mass spectrometry was used to screen proteins that interact with S100A4. **(B)** GO analysis of proteins that interact with S100A4. **(C)** KEGG cluster analysis of proteins that interact with S100A4. **(D)** Co-IP analysis to verify the interaction between S100A4 and GNAI2. **(E)** Packaging of lentivirus overexpressing GNAI2. **(F)** Lentivirus transduction of GNAI2 into endometrial epithelial cells. **(G,H)** Proportion of EdU-positive cells in the GNAI2 overexpression group. **(I,J)** EdU staining-based analysis of the effect of GNAI2 knockdown on cell proliferation.

To determine how S100A4 regulates cell proliferation, we employed mass spectrometry to identify proteins that interact with S100A4. First, we overexpressed S100A4, and after 48 h, the cells were lysed, and the total cellular protein was collected. Protein A/G beads coated with the HA tag antibody were then incubated with the harvested protein to allow binding to the S100A4-HA protein. After the samples were rinsed, we identified the proteins on the resin using mass spectrometry. The results revealed 35 proteins that specifically interact with S100A4 between the overexpression group and the empty vector group (Figure 4A, Supplementary Table S1). Cluster analysis of these proteins revealed that multiple differentially expressed proteins were involved in multiple physiological processes (Figure 4B) and enriched in the “cell growth and death” signaling pathway (Figure 4C), suggesting that interacting proteins downstream of S100A4 may play a role in regulating cell proliferation. We screened proteins that potentially regulate cell proliferation from a set of 35 S100A4-specific enriched proteins. Among them, GNAI2 has been reported to regulate cell proliferation. Therefore, we selected GNAI2 as a target for studying its role in regulating cell proliferation.

To further confirm the interaction between GNAI2 and S100A4, we cotransfected plasmids overexpressing S100A4 and GNAI2 into 293 T cells, with a control group with single plasmid transfection. We collected the protein 48 h after transfection, incubated most of the protein sample with Protein-A/G beads precoated with HA antibody, and used a rinse solution to remove unbound protein. Western blot analysis confirmed the presence of Flag-GNAI2 in the group cotransfected with GNAI2 and S100A4 constructs but not in the other groups (Figure 4D), confirming the interaction between GNAI2 and S100A4.

We then cotransfected the lentiviral vector and viral packaging plasmid into 293 T cells to generate the lentivirus (Figure 4E). The cell supernatant containing the lentivirus was collected, and after endometrial epithelial cells were infected, we observed green fluorescence under a fluorescence microscope 48 h later (Figure 4F). EdU results further demonstrated an increase in cell proliferation after GNAI2 overexpression (Figures 4G,H). To further determine the effect of GNAI2 on cell proliferation, we synthesized a siRNA targeting GNAI2 and transfected the siRNA into endometrial epithelial cells for 24 h. EdU staining also revealed that cell proliferation was suppressed after GNAI2 was knocked down (Figures 4I,J). The above results suggest that GNAI2 can promote the proliferation of endometrial epithelial cells.

### 3.5 GNAI2 regulates the MAPK signaling pathway and interacts with S100A4 to promote cell proliferation

**Figure 5:**
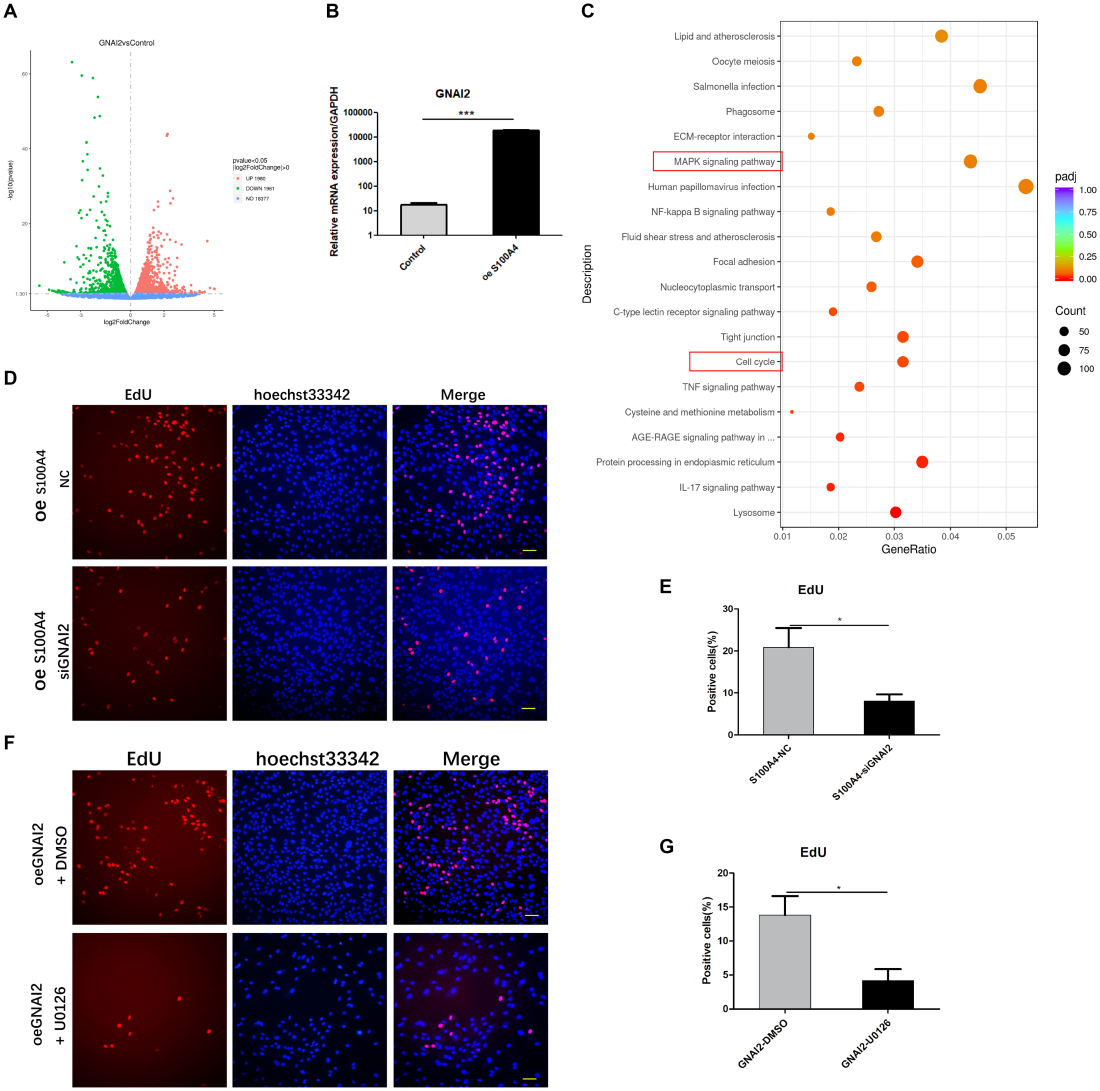
GNAI2 mediates the effects of S100A4 on the promotion of cell proliferation through the MAPK signaling pathway. **(A)** Volcano plot illustrating the transcriptome sequencing results following GNAI2 overexpression. **(B)** qPCR analysis of GNAI2 expression after S100A4 overexpression. **(C)** Cluster analysis of KEGG signaling pathways revealing enrichment of the “MAPK signaling pathway” and “cell cycle” signaling pathways. **(D,E)** EdU staining-based detection of the effect of GNAI2 knockdown on cell proliferation after S100A4 overexpression. **(F,G)** EdU staining-based analysis of the effect of blocking the MAPK signaling pathway on cell proliferation after GNAI2 overexpression.

Given the interaction of GNAI2 with S100A4 and its ability to promote cell proliferation, we investigated whether GNAI2 regulates cell proliferation through a mechanism similar to that of S100A4. We conducted transcriptome sequencing to elucidate the mechanism through which GNAI2 promotes sheep endometrial cell proliferation. We established two groups of endometrial epithelial cells, the empty vector group and the GNAI2 overexpression group, each with three replicates. The results from the volcano plot revealed 1980 differentially expressed genes upon transcriptome sequencing. Among them, 1961 genes were upregulated, and 19 were downregulated following GNAI2 overexpression (Figure 5A). To assess the regulatory effect of S100A4 on GNAI2, we examined the expression level of GNAI2 after S100A4 overexpression using qPCR. The results indicated that overexpression of S100A4 significantly increased the transcription level of GNAI2 (Figure 5B). Cluster analysis of these DEGs revealed enrichment in the “MAPK signaling pathway” and “cell cycle” signaling pathways (Figure 5C), consistent with the findings of transcriptome sequencing analysis after S100A4 knockdown. Furthermore, to clarify whether GNAI2 is involved in the S100A4-mediated regulation of cell proliferation, we knocked down GNAI2 in the cells overexpressing S100A4 and performed EdU staining. The results revealed a notable reduction in the cell proliferation rate (Figures 5D,E). Moreover, to confirm whether GNAI2 regulates sheep endometrial cell proliferation through the MAPK signaling pathway, we treated the cells overexpressing GNAI2 with the MAPK inhibitor U0126 and conducted EdU staining. The results showed that the cell proliferation rate was significantly lower in the treated group than in the control group (Figures 5F,G). These findings suggest that GNAI2 might be a downstream factor in the S100A4-mediated regulation of endometrial epithelial cell proliferation via the MAPK signaling pathway.

### 3.6 Animal experiments verified the downstream regulation of cell proliferation by S100A4

**Figure 6:**
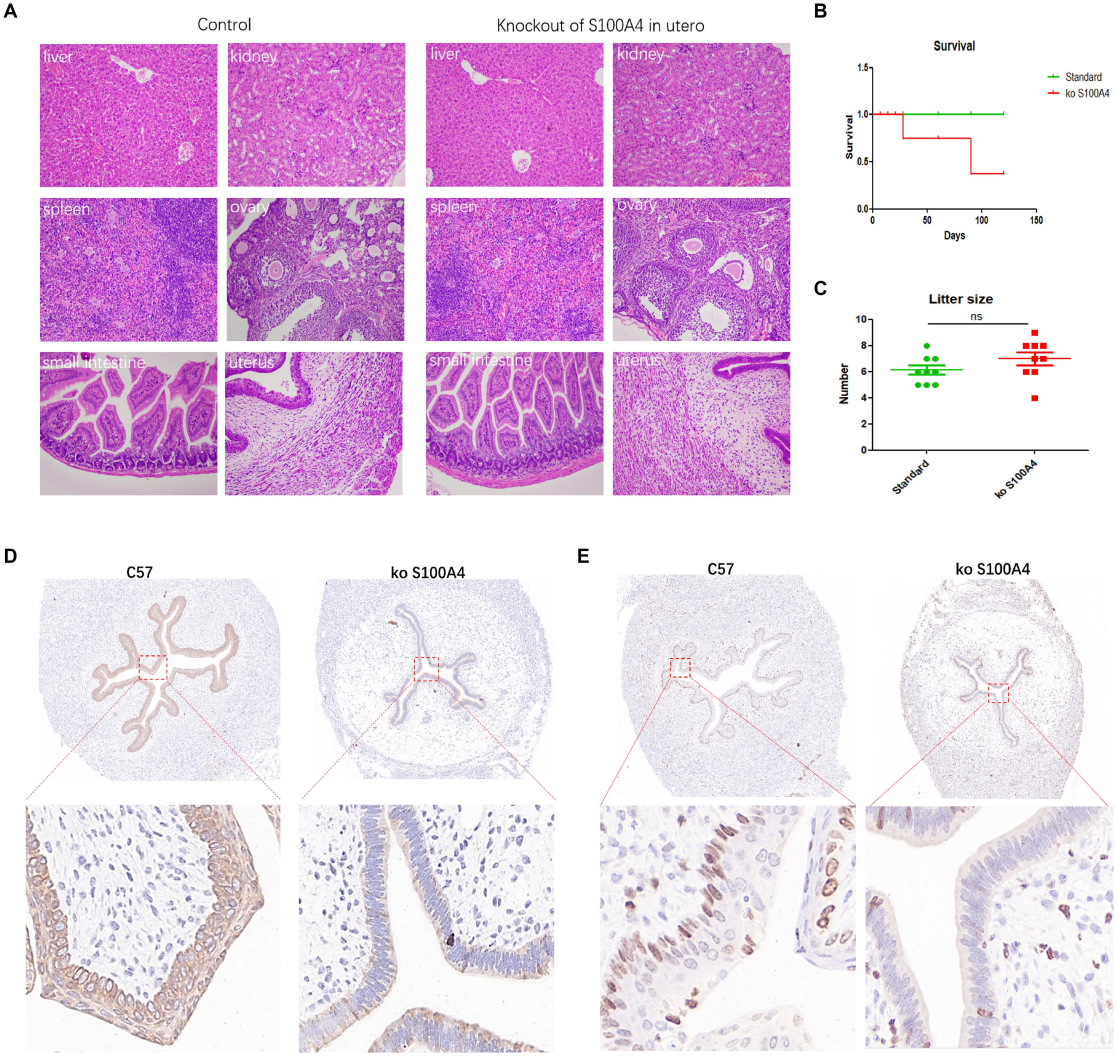
Animal experiments confirming the inhibition of endometrial cell proliferation by S100A4 knockout. **(A)** HE staining results of major organs in the control mice and uterine-specific S100A4 knockout mice. **(B)** Survival curve depicting the impact of S100A4 knockout. **(C)** Statistical analysis of the reproductive parameters. **(D)** Immunohistochemical analysis of p-ERK1/2 expression in the endometrium. **(E)** Immunohistochemical analysis of Ki67 expression in the endometrium.

We employed a crossbreeding approach in which the Pr-Cre enzyme–expressing mice were mated with the conditional S100A4 knockout mice to generate mice with uterine-specific S100A4 deletion. Comparative analysis of the control mice and the mice with uterine-specific S100A4 deletion revealed no significant differences in the main organs. However, several differences in endometrial cell morphology and structure were observed (Figure 6A). In a comparison of the mice with uterine S100A4 deletion to heterozygous mice, one out of ten homozygous mice experienced sudden death before pregnancy but exhibited no other discernible abnormalities (surprisingly, death occurred only when homozygous mice were obtained by crossing heterozygous mice). However, during later rearing and breeding, no abnormal deaths occurred in the offspring of the homozygous mice. The reason is unclear). Another homozygous mouse died unexpectedly after two generations of reproduction, also without typical symptoms. The survival curve is shown in Figure 6B; these results suggest that uterine-specific S100A4 knockout can have systemic effects.

Regarding reproduction, we observed delayed implantation in the mice with uterine-specific deletion of S100A4, but there was no significant difference in the number of offspring per litter (Figure 6C). Preliminary sequencing results indicated that both S100A4 and GNAI2 can regulate downstream MAPK signaling pathways. The key protein node in the MAPK signaling pathway, ERK1/2, becomes phosphorylated (p-ERK1/2) upon pathway activation. Immunohistochemistry was used to determine the expression level of p-ERK1/2. The results demonstrated significantly lower p-ERK1/2 expression in the endometrium of the S100A4 knockout mice than in the endometrium of the control mice (Figure 6D).

To elucidate the impact of S100A4 on endometrial cell proliferation, we employed immunohistochemistry to assess Ki67 expression. The findings revealed a lower number of Ki67-positive cells in the S100A4 knockout group than in the control group (Figure 6E). These findings suggested that S100A4 knockout may inhibit endometrial cell proliferation by suppressing the MAPK signaling pathway.

## 4 Discussion

During embryo transfer, superovulation is often an important factor in regulating reproduction. The most commonly used superovulatory hormone in sheep is FSH (29, 30). The combined use of FSH and eCG can result in increased superovulation (31). GnRH is also widely used for superovulation, and experiments have shown that the combination of GnRH and other hormones has a good effect on superovulation (32). These results suggest that GnRH is effective in promoting superovulation in sheep. However, for embryo transfer in clinical practice, embryo implantation is another factor that affects reproduction. GnRH agonists and inhibitors are widely used in embryo implantation. For example, if a woman who has experienced embryo implantation failure undergoes human embryo transfer, pretreatment with GnRH agonists will increase the live birth rate. However, if women have not experienced embryo implantation failure, pretreatment with GnRH agonists does not increase the live birth rate (33). Similar results were found in patients with uterine fibroids, in which pretreatment with GnRH agonists before embryo transfer increased the live birth rate (34).

Conversely, if GnRH antagonists are used, only high doses will have adverse effects on embryo transfer (35). The effect on embryo implantation, especially at low concentrations, has not been proven. These results suggest that many unknown factors are involved in targeting GnRH to regulate embryo transfer.

In livestock, GnRH also plays a wide range of regulatory roles (36), but some factors are unknown. Five studies analyzed the use of a GnRH agonist versus a control. No significant benefit was demonstrated when GnRH agonists were used (37). Interestingly, these programs did not affect the time interval between calving and conception in dairy cows (38). Beckett and Lean reported that GnRH administration before 40 days after delivery may shorten the time to first estrus but was not associated with the final reproductive performance of the cow (39). A review described the current research on GnRH function in sheep reproduction and indicated that GnRH plays a role in many aspects of reproduction (36). However, there are no direct reports on whether GnRH can regulate embryo implantation or its mechanism. We previously found that treatment with GnRH did not increase the implantation rate of sheep after embryo transfer, but the underlying mechanism is still unclear. We studied the regulatory mechanism involved in endometrial cell proliferation to clarify whether GnRH facilitates sheep embryo transfer. Our results showed that GnRH may inhibit the expression of S100A4 in the endometrium; therefore, GnRH cannot be used during embryo transfer. Endometrial proliferation may also explain why the embryo implantation rate did not increase.

S100A4 has been shown to play an important regulatory role in various diseases and disease models, and its mechanisms of action are relatively diverse. Single-cell sequencing of human tissues revealed that S100A4 is a regulator of immunosuppressive T cells (40). Experiments in mice revealed that the S100A4-mediated increase in mitochondrial metabolism results in a decrease in acetyl-CoA levels, which impairs the transcription of effector genes, especially IFN-γ, facilitating cell survival, tolerance, and memory potential (41). Targeted blockade of S100A4 with an antibody suppressed epithelial-to-mesenchymal transition in asthmatic mice (42). Overexpression of S100A4 also inhibited endoplasmic reticulum stress and ameliorate ischemia–reperfusion injury (43). These findings suggest that S100A4 functions via multiple mechanisms.

The role of S100A4 in regulating proliferation may be most relevant for regulating endometrial receptivity after hormone treatment. Therefore, we performed experiments and found that S100A4 can promote the proliferation of endometrial epithelial cells. In a previous study, S100A4 protein was overexpressed in cultured cancer cells; the results showed that S100A4 protein overexpression did not significantly affect the proliferation of MCF-7 cells but inhibited the proliferation of MDA-MB-231 cells (44). Similarly, S100A4 was found to regulate cell migration but not cell proliferation in gastric adenocarcinoma (20). However, when S100A4 targets leukemia cells, it also promotes cell proliferation (45). In experiments on PC3 prostate cancer cells, intracellular S100A4 expression was found to be positively correlated with cell proliferation, revealing that S100A4 promotes cancer cell proliferation (46). The effect of S100A4 on cell proliferation may be affected by the cell type and the localization of S100A4.

We sequenced the transcriptome of endometrial epithelial cells with S100A4 knockdown, and subsequent Gene Ontology (GO) enrichment analysis of the DEGs revealed that S100A4 plays a role in cell proliferation-related mechanisms. KEGG enrichment analysis revealed enrichment of the MAPK signaling pathway. This pathway is important for regulating the proliferation of various cell types (47, 48). MAPK activation can promote cell proliferation. Sheep basic fibroblast growth factor (bFGF), epidermal growth factor (EGF), and vascular endothelial growth factor (VEGF) can quickly activate the MAPK signaling pathway to regulate cell proliferation (49). Several biologically active substances, such as osteocalcin (50), progesterone and FSH (51), and thyroid hormone (52), can target the MAPK signaling pathway to regulate cell proliferation. Similarly, GnRH can also regulate the MAPK signaling pathway to regulate cell proliferation (53). These findings suggested that S100A4 may regulate the proliferation of endometrial epithelial cells by activating the MAPK signaling pathway.

GNAI2, which belongs to the G protein family, is one of the key genes involved in melanogenesis (54). Studies have shown that GNAI2 can promote cell proliferation (54, 55). However, no studies have shown whether GNAI2 can promote cell proliferation, and its mechanism of action in endometrial cells is unclear. In our experiments, we found that S100A4 can interact with GNAI2 and that GNAI2 can promote endometrial cell proliferation, which expands the known list of targets of S100A4.

Various cells can secrete the S100A4 protein (56–58); this finding may also explain why mice can still reproduce normally after S100A4 is knocked out of the uterus. However, in the case of uterine-specific S100A4 knockout, some events require special consideration, such as the sudden death of mice, but the explanation is unclear. In addition, the proliferation of endometrial cells was significantly inhibited, and the expression of p-ERK1/2 was significantly reduced in the mice with uterine-specific S100A4 knockout. This result is similar to the results of previous cell experiments. We speculated that in animals, S100A4 can target the MAPK-ERK1/2 signaling pathway to regulate endometrial cell proliferation.

## 5 Conclusion

While GnRH is widely utilized in animal reproduction, particularly in practical operations such as embryo transfer to promote animal quality, our previous investigations have not conclusively demonstrated its efficacy in promoting embryo implantation. Furthermore, the underlying mechanism of action remains unclear. During our research, we discovered that the application of GnRH may actually impede the proliferation of endometrial cells through the S100A4-GNAI2-MAPK signaling pathway, which has adverse effects on embryo implantation. Our findings elucidated a previously unrecognized aspect of the mechanism by which GnRH regulates endometrial cell proliferation. These findings can potentially offer valuable guidance for optimizing hormone treatment in the context of animal reproduction.

## Data availability statement

The original contributions presented in the study are publicly available. These data can be found in the NCBI BioProject repository: (1) https://www.ncbi.nlm.nih.gov/bioproject/PRJNA1090558. (2) https://www.ncbi.nlm.nih.gov/bioproject/PRJNA1090239.

## Ethics statement

The animal studies were approved by the Ethical Committee of Xinxiang Medical University. The studies were conducted in accordance with the local legislation and institutional requirements. Written informed consent was obtained from the owners for the participation of their animals in this study.

## Author contributions

XJ: Conceptualization, Data curation, Formal analysis, Investigation, Methodology, Software, Validation, Writing – original draft, Writing – review & editing. ZC: Conceptualization, Funding acquisition, Supervision, Visualization, Writing – original draft. ML: Data curation, Methodology, Writing – original draft. JW: Data curation, Methodology, Writing – original draft. ZR: Data curation, Methodology, Writing – original draft. LW: Data curation, Methodology, Writing – original draft. CL: Data curation, Methodology, Writing – original draft. XL: Writing – review & editing. FR: Funding acquisition, Writing – review & editing. XW: Conceptualization, Funding acquisition, Project administration, Resources, Supervision, Writing – review & editing.
