# Corrigendum: GnRH-mediated suppression of S100A4 expression inhibits endometrial epithelial cell proliferation in sheep via GNAI2/MAPK signaling

**DOI:** 10.3389/fvets.2024.1445291

**Published:** 2024-08-20

**Authors:** Xiyao Jiao, Zhili Chu, Meng Li, Jiurong Wang, Zilong Ren, Leyang Wang, Chengcheng Lu, Xiangyun Li, Feng Ren, Xinglong Wu

**Affiliations:** ^1^College of Animal Science and Technology, Hebei Technology Innovation Center of Cattle and Sheep Embryo, Hebei Agricultural University, Baoding, China; ^2^School of Basic Medical Sciences, Xinxiang Medical University, Xinxiang, China; ^3^Henan International Joint Laboratory of Immunity and Targeted Therapy for Liver-Intestinal Tumors, Xinxiang Medical University, Xinxiang, China

**Keywords:** sheep endometrial epithelial cells, GnRH, cell proliferation, S100A4, GNAI2, MAPK signaling pathway

In the published article, there was an error in Figure 1 as published. In Figure 1C, the GFP section of GnRHR and S100A4 are placed in the wrong position, a mistake owing to our negligence in combining these figures. The corrected Figure 1 and its caption appear below.

**Figure 1:**
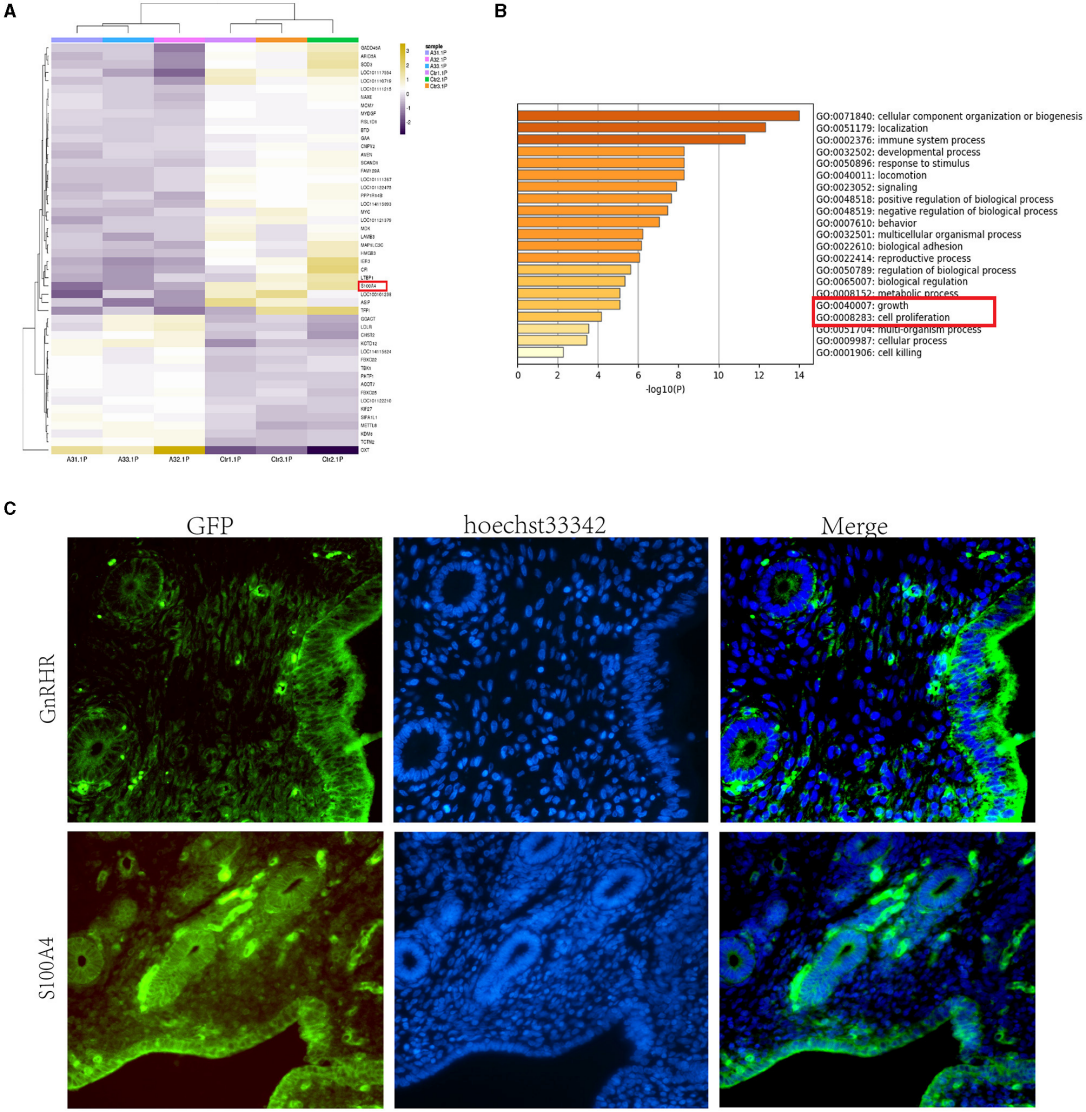
GnRH reduces the expression of S100A4 in the sheep endometrium. **(A)** Heatmap showing significant variation in the expression level of S100A4. **(B)** Transcriptome sequencing and GO analysis revealed that GnRH affects cell proliferation-related signaling pathways, such as “growth” and “cell proliferation,” in treated sheep. **(C)** Expression of GnRHR and S100A4 in the sheep endometrium; the results show that the localization patterns of S100A4 and GnRHR are similar.

The authors apologize for this error and state that this does not change the scientific conclusions of the article in any way. The original article has been updated.

