# Supplementary material for: GnRH-mediated suppression of S100A4 expression inhibits endometrial epithelial cell proliferation in sheep via GNAI2/MAPK signaling
Source: Front Vet Sci. 2024 May 30;11:1410371. doi: 10.3389/fvets.2024.1410371 (PMC11169792; doi:10.3389/fvets.2024.1410371)
Supplement: Supplementary file 1 [file Data_Sheet_1.PDF]

Identified by	Total	Accession	Names	Sequence
Ctrl	68	tr|W5NVY0|W5N	Uncharacterized	MGGSDRGGFN
		tr|W5NUI0|W5NU	Tubulin beta chai	KYVPRAALVDLE
		tr|W5Q0L1|W5Q	Elongation factor	MRHATTTSYPT
		tr|W5QCY8|W5Q	Hemoglobin subu	TMLTAEEKAAVT
		tr|W5P5T8|W5P5	40S ribosomal pr	SAAPQALLELE
		tr|W5PJY3|W5PJ	60S ribosomal pr	MSKRGRGGSS
		tr|W5NPI6|W5NP	Polyadenylate-bir	AERALDTMNFD
		tr|W5Q0Q1|W5Q	Tyrosine 3-mono	SAPPAHMEKTE
		tr|W5Q000|W5Q	Ribosomal protei	MAALRPLVKPKI
		tr|W5Q4E3|W5Q	ATP-dependent	MGVMPEIAQAV
		tr|W5P010|W5P0	RAB1B, member	VRPARAVRRSL
		tr|W5P9P0|W5P	Unconventional n	MEDGRPVWAP
		tr|W5PCR3|W5P	Scaffold attachm	MAESLSGLGDS
		tr|W5PH41|W5P	Smoothelin OS=	MADETLAGLDE
		tr|W5Q7Z7|W5Q	Desmoplakin OS	FSQTGTMSRHQ
		tr|W5PIG7|W5PI	2-phospho-D-gly	MSILKVHAREIF
		tr|W5QBS6|W5Q	Actin-related prot	ASTDQSPRIQR
		tr|W5PPT7|W5P	40S ribosomal pr	MLMPKKNRIAYI
		tr|W5P2U9|W5P	Leucine rich repe	DERLLSASLAR
		tr|W5Q4T1|W5Q	LRR binding FLII	MGTPGSGRKRT
		tr|W5NUV1|W5N	G protein subunit	MSELDQLRQEA
		tr|W5P0Z0|W5P	Ribosomal protei	MSSKVSGDTLY
		tr|W5PUU0|W5P	60S ribosomal pr	MAPAKKGGEKK
		tr|W5PUT8|W5P	Actin-related prot	MARNTLSSRFR
		tr|W5PBP1|W5P	Ribosomal protei	GGVWGRRSEM
		tr|W5PK00|W5P	Heterogeneous n	MMLSTEGREGF
		tr|W5PKR5|W5P	Family with seque	MECDLMETDILE
		tr|W5Q6A4|W5Q	Keratin, type I cyt	MTSYSYRQSSS
		tr|W5PCM4|W5P	Leucine zipper pr	MAEFTSYKDTA
		tr|W5PRF0|W5P	Activated RNA pc	MPKSKELVSSS
		tr|W5Q0K9|W5Q	Actin binding LIM	MDPHHILEDHR
		tr|W5PED1|W5P	DNA ligase OS=	MTLAFKILFPPTL
		tr|W5PT68|W5P	Filamin B OS=Ov	MPVTEKDLAED
		tr|W5P5S7|W5P	PDZ and LIM dor	MDSFKVVLEGP
		tr|W5QAZ1|W5Q	Coronin OS=Ovis	MTVTKMSWRP
		tr|W5PWA8|W5P	Heat shock protei	MAERRVPFSLLF
		tr|W5NXM4|W5N	40S ribosomal pr	MPVARSWVCR
		tr|W5Q0A5|W5Q	DEAD box protei	XHFHRSLVGQP
		tr|W5Q2S8|W5Q	Myosin light chair	MLLRIKGDKARK
		tr|W5QI04|W5QI	Tropomyosin 1 O	QQPASPTAARP
		tr|W5QFL1|W5QI	Tubulin alpha cha	MRECISVHVGQ
		tr|W5QFP4|W5Q	Histone H4 OS=	SLIMSGRGKGG
		tr|W5QIF5|W5QI	cAMP regulated	MSAEVPEAASA
		tr|W5PAX2|W5P	SH3 domain bind	VDVILCLDLAGS
		tr|W5PFM2|W5P	Adaptor related p	MIHSLFLINCSG
		tr|W5NVB9|W5N	Actin-related prot	MSNNTVSSARF
		tr|W5PUU6|W5P	60S ribosomal pr	QAKIKARDLRGK
		tr|W5PTW2|W5P	Phosphate carrie	MYSSVVHLARA
		tr|W5QFP1|W5Q	Polyadenylate-bir	MNAAASSYPMA

		tr|W5QBE7|W5Q	Ribosomal protei	MASVSELACIYS
		tr|W5PDR4|W5PI	BARX homeobox	TARAHPAPHSLF
		tr|W5PH51|W5PH	40S ribosomal pr	QSLVIPEKFQHIL
		tr|W5PCQ8|W5P	ADP/ATP translo	MSDQALSFLKDF
		tr|W5PW68|W5P	Uncharacterized	EVVIPQKKGKKA
		tr|W5PDV0|W5PI	Sorbin and SH3 c	MFRFLDDDTDM
		tr|W5QAX3|W5Q	Actin gamma 1 O	IAMEEEIAALVIDI
		tr|W5QDT2|W5Q	Splicing factor pr	MSRDRFRSRGG
		tr|W5QD94|W5Q	G protein subunit	MGSGASAEDKE
		tr|W5PEV6|W5PE	40S ribosomal pr	SLSPCWQRGGF
		tr|A8DR93|A8DR	Heat shock protei	MPEETQAQDQP
		tr|W5PDF4|W5PI	Plakophilin 3 OS=	SVRSLSLGDSGI
		tr|W5QAY7|W5Q	Ribosomal protei	YRVELCSFSGYK
		tr|W5Q6L2|W5Q	60S ribosomal pr	LKMVRYSLDPEN
		tr|W5NV97|W5N	Uncharacterized	MVEESGTLESQI
		tr|W5NYB8|W5N	Uncharacterized	PSPFRLRLLSVE
		tr|W5PVA8|W5P	40S ribosomal pr	GGSGCSCSPGF
		tr|W5PPH6|W5PI	40S ribosomal pr	MSAHRRRRRAV
		tr|W5NY50|W5N	ATP synthase su	MLSVRVAAAVAF
Treat	35	tr|W5PNV2|W5PI	Serine/threonine-	MSDSEKLNLDSI
		tr|W5QIF6|W5QI	cAMP regulated	AEHKMKTTFARV
		tr|W5Q4T0|W5Q	LRR binding FLII	MGTPGSGRKRT
		tr|W5QAW7|W5Q	Histone H2B OS=	MPEPAKSAPAPK
		tr|W5Q5K4|W5Q	Ribosomal protei	MSSKVSRDTLYE
		tr|W5PKD3|W5PI	G protein subunit	MGCTVSAEDKA
		tr|W5QF03|W5QI	40S ribosomal pr	MKLNISFPATGC
		tr|W5P9P2|W5P9	Unconventional m	MEDGRPVWAPH
		tr|W5NR63|W5NI	Protein S100 OS=	PRRSPHQPTIGC
		tr|W5QFL9|W5QI	Histone H2A OS=	AAVEFEMSGRG
		tr|W5PMJ5|W5PI	RNA binding moti	KMVEADRPGKLI
		tr|W5Q438|W5Q	G protein subunit	PLPGRASAPPLF
		tr|W5QFN8|W5Q	Histone H4 OS=C	KEQQLLFIMSGR
		tr|W5P627|W5P6	Actin-depolymeriz	MLPKRPSSPPRS
		tr|W5P4R1|W5P4	Moesin OS=Ovis	MVGGRQRKVKD
		tr|W5PJU6|W5PJ	Phenylalanine--tR	MATQARPTPQS
		tr|W5NUM4|W5N	Oxysterol binding	MKEEAFLRRRFS
		tr|J9SVG9|J9SVG	High glycine/tyros	MCGYYGNYYGG
		tr|W5Q6P5|W5Q	Hair keratin type	MTCGSYRAVPA
		tr|W5PDQ3|W5P	Scaffold attachme	MAETLAGSSDSG
		tr|W5QIH5|W5QI	Tropomodulin 2 C	MALPFQKELEKY
		tr|W5PNS4|W5PI	60S ribosomal pr	MPDGQVPHEGS
		tr|W5NT34|W5N	BAF nuclear asse	MTTSQKHRDFV
		tr|W5PDG3|W5P	Glyceraldehyde-3	MVKVGVNGFGR
		tr|W5Q3X9|W5Q	Keratin 33B OS=	MSYNFCLPNLSF
		tr|W5P1G7|W5P1	Heterogeneous n	MMLGPEGGEGF
		sp|P15241|K2M2_	Keratin, type II m	CGFSTVGSGFG
		tr|W5NS65|W5NS	Histone H1.4-like	MSEAVPAAPPAI
		tr|W5QHQ7|W5Q	Nucleolin OS=Ov	AGKNQGDSKKM
		tr|W5P7Y1|W5P7	MLVIN_C domair	TIPSHNIGPGDW
		tr|W5NR85|W5NI	Keratin associate	TMSYCFSSTVFF
		tr|W5NSS8|W5N	Tubulin beta chai	QFWEVISDEHGI

		tr|W5PYZ3|W5PY	Actin-related prot	ASTDQSPRIQRS
		tr|W5NRP7|W5N	Keratin-associate	MSYNCCSGNFS
		tr|W5NY24|W5NY	Catenin beta 1 O	MATQVADLMELI
Ctrl	107	tr|W5P640|W5P6	Lamin A/C OS=O	METPSQRRATR
Treat				
		tr|W5PJ75|W5PJ	Spectrin alpha, n	MDPSGVKVLETA
		tr|W5QBL7|W5QI	Actin-related prot	MAYHSFLVEPIS
		tr|W5Q096|W5Q0	Uncharacterized	CLDEYEDDEAG
		tr|W5NZX9|W5NZ	Spectrin beta cha	MTTTVATDYDNI
		tr|W5QIH2|W5QI	Tropomodulin-3 (	MALSFRKDLEKY
		tr|W5PX97|W5PX	Myosin IB OS=O	MAKMEVKTSLLD
		tr|W5PZS4|W5PZ	Oxysterol-binding	MSQRQGKEAFP
		tr|W5P824|W5P8	Actin-related prot	QTATLRPYLSAV
		tr|W5NPQ4|W5N	F-actin-capping p	MDQKSKCKLRR
		tr|W5QDZ3|W5Q	Arp2/3 complex 3	GSAGGFRLQGP
		tr|W5P6P4|W5P6	Ribosomal protei	FLSAVVREKKKK
		tr|W5Q6V2|W5Q6	Keratin 7 OS=Ov	MSLHFGSQVFSS
		tr|W5Q5M3|W5Q	Keratin 18 OS=O	SPGLALSSRTNII
		tr|W5NRW4|W5N	Tropomyosin 4 O	MEAIKKKMQMLK
		tr|W5Q8V2|W5Q8	LIM domain and a	MEPTPFNRRQW
		tr|W5PNW7|W5P	Vimentin OS=Ovi	PSAAMSTRSVSS
		sp|A2SW69|ANX	Annexin A2 OS=(	MSTVHEILCKLSI
		tr|W5PY60|W5PY	Cytospin-A OS=C	MKRATRSVGSA
		tr|W5NRR3|W5N	Poly [ADP-ribose]	MAESSDKLYRVE
		tr|W5NU63|W5NU	Myosin heavy cha	MAQRTGLEDPE
		tr|W5PK94|W5PK	Myosin ID OS=O	SFLPLVCRFEKG
		tr|W5PQC5|W5P	60S acidic riboso	MPREDRATWKS
		tr|W5PHA3|W5PI	Tr-type G domain	MGKEKTHINIVVI

tr|W5PBM0|W5PI	60S ribosomal pr	LQQDQGEKENP
tr|W5QC37|W5Q	Tubulin alpha cha	MRECISIHVGQA
tr|W5PQR5|W5P	Ribosomal protei	MGVDIRHNKDRI
tr|W5PC98|W5P(	Synaptopodin OS	MLGPHLPSLPPA
tr|W5NWP4|W5N	RNA helicase OS	MSHVAVENALGI
tr|W5PCT5|W5P(	ADP/ATP translo	QVQHASKQIAAD
tr|W5PSW9|W5P	60S ribosomal pr	MGRVIRGQRKG
tr|W5PX99|W5P>	Myosin IB OS=O	MAKMEVKTSLLD
sp|Q9MZA9|VIME	Vimentin (Fragme	MSTRSVSSSSYI
tr|W5Q8J9|W5Q8	40S ribosomal pr	ARGPKKHLKRV/
tr|W5P4L7|W5P4	Actin binding LIM	MNTSLPYQQNP
tr|W5PHT8|W5PI	Dedicator of cytol	CGESRSFYFKLS
tr|W5PN93|W5PI	Coronin OS=Ovis	MSWNPQYRSSK
tr|W5Q4W8|W5Q	75 kDa glucose-r	MISASRAAVSRL
tr|W5Q2U7|W5Q	Plectin OS=Ovis	MVAGMFMPLDQ
tr|W5PE92|W5PE	Granulin precurs	MWTLVSWVALV
tr|W5NUU3|W5N	Tropomyosin 3 O	RMEAIKKKMQMI
tr|W5Q3U3|W5Q	Galectin OS=Ovis	MAFGGAQASYIN
tr|W5P7P6|W5P7	Myosin XVIIIA OS	MFNLMKKDKDK
tr|W5PK33|W5PI	EF-hand domain	TESGGWVSTAS
tr|W5PLW1|W5P	Catenin delta 1 O	MDDSEVESPASI
tr|W5Q740|W5Q	ATP binding cass	LSIFCELYLVRGS
tr|W5Q7R8|W5Q	Junction plakoglo	ATMEVMNLIEQF
tr|W5PQ47|W5P(	Retinoic acid indu	MCKLASWSLKV
tr|W5PUT6|W5PU	Clathrin heavy ch	YNLPVIFSFLKSS
tr|W5Q2P8|W5Q	LIM domain 7 OS	MEEAEADCALAI

tr|W5Q951|W5Q9	Coronin OS=Ovis	MEGPGQLGRSG
tr|W5QIG1|W5QI	Myosin VC OS=C	AICILLLNLGQYN
tr|W5PK27|W5PK	Myosin light chair	MPPKKDVPVKKI
tr|W5NSP2|W5NS	40S ribosomal pro	RAAFSLSSQRRA
tr|W5Q0B6|W5Q0	Protein phosphata	MKMADAKQKRN
tr|W5Q6N9|W5Q	AP-3 complex sul	MKFFFFVYRNED
tr|W5Q4S3|W5Q4	Ribosomal protei	MSHRKFSAPRH
tr|W5QAK6|W5Q	ATP-dependent D	VSSNNMSGWES
tr|W5PWH2|W5P	Myosin heavy cha	MAAVTVSVPGRI
tr|W5P3J0|W5P3	60S ribosomal pro	MAGEKAEKPDTI
tr|W5PN88|W5PN	Eukaryotic transla	QVNFTVDQIRAII
tr|W5PUS9|W5PU	Ribosomal protei	MGAYKYIQELWI
tr|W5NZ21|W5NZ	ATPase family AA	MSWLFGIKGSKG
tr|W5QJ62|W5QJ	Actinin alpha 1 O	ALFDPVRCLNQG
tr|W5PPT6|W5PP	Tubulin beta chai	MREIVHIQAGQC
tr|W5PJ98|W5PJ	F-actin-capping p	VPSLCEDLLSSV
tr|W5Q5C7|W5Q	Catenin alpha 1 O	MTAVHTGNINFK
tr|W5PHW0|W5P	Heat shock protei	MPEEVHHGEEE
tr|W5PSS9|W5PS	60S ribosomal pro	MTNTKGKRRGT
tr|W5QFG8|W5Q	Actin related prot	LESAAKLFIFVDG
tr|W5PUV3|W5PU	Ecto-5'-nucleotida	MDPRAARTPALI
tr|W5NQM5|W5N	Cortactin OS=Ovi	FQNDVSEKEQR
tr|W5PQ98|W5PQ	Actin-like protein	YTKLGYAGNTEI
tr|W5PG95|W5PG	Heat shock 70kD	TGMAKNTAIGID
tr|W5Q5N9|W5Q	Keratin 8 OS=Ovi	MSIRVTQKSYKV

tr|W5PYH6|W5P	AP-3 complex su	MALKMVKGSIDI
tr|W5Q804|W5Q	Sperm antigen wi	SGAVTATKRTGI
tr|W5QJ98|W5Q	Calmodulin-alpha	MADQLTEEQIAE
tr|W5Q5K9|W5Q	Actin-related prot	SVLVCPSDICSL
tr|W5QGC1|W5Q	Leucine rich repe	MAAAGLVAVAA
tr|C5ISB1|C5ISB	Replication protei	MVGHLSEGAIA
tr|W5P114|W5P1	Ribosomal protei	MTEWETAAPAV
tr|W5PF97|W5PF	Ribosomal_L18_	MGFVKVVKNKA
tr|W5NW47|W5N	Myosin_tail_1 do	MRKKMDDSVG
tr|W5NPN4|W5N	Heat shock prote	MSKGPAVGIDL
tr|W5QBQ9|W5Q	Myosin heavy ch	MAQQAADKYLY
tr|W5PL19|W5PL	Myosin regulator	MSSKKAKTKTK
tr|W5QI03|W5QI	Tropomyosin 1 O	MDAIKKKMQML
tr|W5QI00|W5QI	Lactamase beta	DEVGAPGIVVG
tr|W5QDK7|W5Q	X-ray repair cros	MARSWNKAAV
tr|W5Q675|W5Q	40S ribosomal pr	PPVALGAESHLI
tr|W5P7Z1|W5P7	Uncharacterized	AAPRGEDLVSTI
tr|W5PSC5|W5P	Myosin IC OS=O	GRMALQVELIPT
tr|W5P1D8|W5P1	Heterogeneous n	MAAGVEAAAEV
tr|W5PTR5|W5P	78 kDa glucose-r	KMKLSLVAAVLL
tr|W5NVZ1|W5N	Myosin phosphat	AKPIYGGWLLLA
tr|W5Q6B8|W5Q	Keratin 6A OS=O	TMSYKSTVKTQ
tr|W5QEH0|W5Q	Twinfilin actin bi	VSLRSRVXEER
tr|W5QFZ8|W5Q	60S ribosomal pr	MAEGQVLVLDG
tr|W5P5J0|W5P5	Drebrin 1 OS=Ov	ALYTYEDGSDD
tr|W5P0H3|W5P	60S ribosomal pr	MPKGKKAKGKK

tr|W5P707|W5P7	Actinin alpha 4 O	MAQEDDWDRDI
tr|W5Q9Z9|W5Q9	60S ribosomal pr	MMFLNINLFFYFI
tr|W5PY97|W5PY	Supervillin OS=O	MKRKERIARRLE
tr|W5NR09|W5NI	FLII actin remode	MSQTSLTVTYPC
sp|P60713|ACTB_	Actin, cytoplasmic	MDDDIAALVVDN
tr|W5Q694|W5Q6	F-actin-capping p	MADFEDRVSDE

Length	Mass	Unused	Coverage(%)	Unique PepSeq
253	26200.3	2	3.556999937	AAIDWFDGK
373	41908.1	2.14	23.05999994	YLTVAAIFR,MSA
558	63420.3	2	2.329999954	ALIAAQYSGAQV
146	16174.5	4	15.07000029	LLVVYPWTQR,L
174	19886.2	2.04	6.897000223	AIIIFVPVPQLK
140	14865.3	2	14.2900005	ISLGLPVGAVINC
572	63535.3	6	6.993000209	GFGFVSFER
251	28305.4	2	4.780999944	SICTTVLELLDK
135	15873.7	2	9.629999846	SYCAEIAHNVSS
736	82003.2	3.25	4.075999931	GIDIHGVPYVINV
307	32912.1	2	3.582999855	LLLIGDSGVGK
1285	148635.8	20.93	12.06	AGSLKDPLLDDH
900	100493.9	2	1.111000031	ILDILGETCK
886	96112.1	2.02	1.353999972	AQEIEAATLAGR
2748	317876.5	2.07	0.509499991	YCYLQNEVFGLF
443	48195.6	2	3.386000171	VNQIGSVTESLQ
403	45231.2	6.15	21.59000039	TLESSIQGLR,SL
165	18861.6	6.72	23.63999933	AEAGAGSATEF(
330	37349.8	3.12	13.03000003	SHDILQWVLQTD
655	73879.1	10.48	11.15000024	LKLQLEEER,FSA
340	37376.6	2	3.234999999	LIIWDSYTTNK
217	24639	3.03	13.35999966	KYDAFLASESLIK
125	14462.8	2	11.20000035	LYTLVTYVPVTTI
160	17677.2	5.23	20.0000003	AFHAALR,ALAV(
160	17257.7	2.12	8.124999702	IEDVTPIPSDSTF
449	49249.3	2	3.785999864	STGEAFVQFAS(
519	55446.6	2.09	1.926999912	LCAWLVSELR
458	49615.6	2.01	10.70000008	FGAGGAFR
1038	114992.4	2.48	0.963400025	RLDELEEATK
127	14395.2	2.21	10.23999974	GISLNPEQWSQI
781	87985.8	3.93	3.584999964	FPAAQAPDPSEI
1010	113525	5.36	4.554000124	EGLEGLVLK,HVI
2640	282407.3	5.28	1.590999961	VLFASQEIPTSPI
584	63370.1	2.01	2.054999955	VVLEGPAPWGF
480	54919	2.02	2.707999945	VLIWNLDVGEPV
184	19508.8	2	5.434999987	LFDQAFGLPR
194	22682.3	2.28	5.669999868	ELLTLDEKDPR
669	75343.7	5.5	5.082000047	GDGPICLVLAPT
312	34987.1	4.5	22.43999988	NAFACFDEEAS(
278	31555.7	2.29	40.29000103	CAELEEELKTVT
452	50282.4	15.51	25.44000149	TIQFVDWCPTGI
106	11680.7	4	18.87000054	VFLENVIR,DNIQ
1883	218504.2	6.96	3.877000138	LGILDLLDEECK,
117	13505.2	2	10.26000008	ESNTVFSFLGLK
418	46938.8	2.87	5.262999982	VLSFIPPDGNFR
170	18295.7	2	7.058999687	ALAAGGVGSIVF
107	12498.9	2.09	12.15000004	QLEDLKVELSQL
361	39987.3	2	2.215999924	FGFYEVFK
660	72312	2	7.272999734	SLGYAYVNFQQI

114	11513.8	2	14.04000074	AAGVNVEPFWP
193	20767.7	2	8.29000026	NSIPTSEQLTEQ
152	17715.5	10.44	32.24000037	YSQVLANGLDN
299	32911.1	2	8.360999823	DFLAGGVAAAIS
592	64587.1	2.03	2.364999987	GFGFVDFNSEE
468	52676.1	5.74	6.409999728	KGDTVYILR,FF
377	41976.7	55.36	75.33000112	MEEEIAALVIDN
654	73376.5	2	2.598999999	LFVGNLPADITE
357	40398.8	2.03	3.081000037	LLLLGAGESGK
157	17389.9	4.88	14.65000063	ELAPYDENWFY
733	84730	2	3.54700014	ADLINNLGTIAK
585	64029.7	2.01	2.563999966	LSSGFDDIDLPS
157	17838.8	2	5.731999874	QINWTVLYR
186	21629.2	2.02	5.375999957	SAEFLLHMLK
239	27617.7	2	5.438999832	ELPTAFDYVEFT
454	47282.8	2.07	1.542000007	LNLWISR
137	14753.1	4.69	15.33000022	LNNLVLFDK,DK
281	31046.3	9.39	21.34999931	GCEVVVSGK,GL
553	59703	2.48	5.424999818	HALIIYDDLSK,F
330	37511.7	4.17	12.42000014	AHQVVEDGYEF
1854	215067.5	22.04	9.76300016	LTNLEGTYNSET
721	81631.9	3.11	5.824999884	DRFSAEDEALS
141	15509.9	2.37	10.63999981	AMGIMNSFVND
217	24793.1	2	5.990999937	KYDAFLASESLI
355	40476.6	3.51	11.82999983	IAQSDYIPTQQD
206	23674.7	1.38	9.708999842	LNISFPATGCQK
1254	145104.8	14.7	8.692000061	HFAGAVCYETT
126	14112.1	1.46	15.08000046	ALDVMVSTFHK,
134	14552.8	2.57	14.18000013	VTIAQGGVLPNI
392	42455.6	2.2	3.57099995	GFAFVTFESPA
358	38876.5	2.13	6.98300004	LIIWDSYTTNK,S
111	12367.5	4.47	40.5400008	DNIQGITKPAIR,
782	85726.8	3.09	3.325000033	TPSAAYLWVGA
665	77511.2	1.3	1.353000011	IGFPWSEIR
542	60935.9	2	2.583000064	SLQALGQIIETEL
516	55997.3	1.48	6.395000219	GESVGSITQPLP
71	7285	2	19.72000003	LGCGYGCGYGY
461	50621.9	19.97	26.24999881	LSSELNSLQEVL
915	102959.5	2.03	2.185999975	EEKMEKKEEK,IL
383	43116.8	4.34	6.789000332	DREDFVPFTGE
135	14582.9	2.84	12.59000003	TIGISVDPR,GFS
89	10058.5	2.09	13.4800002	AYVVLGQFLVLK
334	36036.2	7.93	20.96000016	VIHDHFGIVEGL
408	46290.2	12.67	22.30000049	TVNALEVELQA
403	44929.1	2	4.218000174	ITGEAFVQFASQ
491	53681.3	2.16	13.03000003	GLTGGFGSR
221	22633.9	2	4.977000132	ALAAAGYDVEK
677	73338.9	2.45	2.067999914	GFGFVDFNSEE
369	42170.6	2	4.064999893	REGLDFWLILG
63	6891.6	1.6	12.70000041	RFWPFALY
427	47985.5	2	25.06000102	MSATFIGNSTAI

401	45000.9	17.11	39.39999938	FCTTGIDGAMTI
164	17472	2	7.316999882	SSFYRPTFFSSR
782	85609.1	2	2.813000046	NEGVATYAAAVL
658	73618.1	1.37	2.735999972	LADALQELR
		1.37	2.735999972	LADALQELR
2257	259913.5	10.07	3.367000073	HQAFEAELSAN
		7.24	2.923999913	AGTFQAFEQFG
364	40272.7	9.36	20.88000029	FAVGSGSR,TW
		6.79	19.23000067	AYHSFLVEPISC
168	19417.2	3.54	17.26000011	STTTASEEDVS
		4.02	24.40000027	QEPSRTVSGRY
2363	274403.8	4.17	2.157999948	HQILEQAVEDYA
		2.2	0.465499982	FMELLEPLNER
352	39661.5	11.9	25.56999922	ILPVFDEPPNPT
		18.07	36.0799998	SNDPVAIAFADM
1138	132030	4	41.11999869	CEEAVTTIAAYW
		3.09	38.65999877	CEEAVTTIAAYW
848	97115.4	4.29	2.94799991	VVLPTFILEPR,L
		7	7.19299987	FYGNSLSAILEG
168	19615.7	4.93	16.06999934	AENFFILR,IVAE
		2.2	4.761999846	AENFFILR
313	36330.8	8.94	26.51999891	EGAAHAFAQYN
		14.12	53.67000103	EHYPNGVCTVY
375	41728	11.71	15.46999961	DNTINLIHTFR,Y
		11.04	15.72999954	ELQAHGADELL
250	29423.9	6.26	14.00000006	IALTDNALIAR,E
		6.25	14.00000006	IVEPYIAWGYPN
452	50180	15.32	33.84999931	LQAEIDNIK,VDG
		9.92	27.4300009	LALDIEIATYR,A
409	46047.2	17.41	29.10000086	GLQNQIANSGLT
		7.95	17.84999967	GLQNQIANSGLT
284	32751.4	3.9	33.10000002	LVILEGELER,AE
		13.75	42.96000004	LVILEGELER,KA
760	85087.1	37.73	37.63000071	LSLGTYASLHGF
		36.51	36.57999933	RQWASLSLR,HF
467	53686.2	84.7	69.5900023	KVESLQEEIAFL
		59.78	67.44999886	KVESLQEEIAFL
339	38611.7	6.18	12.08999977	GVDEVTIVNILTN
		11.68	22.41999954	TNQELQEINR,G
1094	120633.4	15.47	9.414999932	ETIFELEDEVEQ
		16.61	10.23999974	LNALGFSLEQR,
1016	113354.5	13.95	10.72999984	ILTLGK,SLQELL
		12.87	13.87999952	LQLLEDDKESR,
1976	229098.4	216.52	61.94000244	LVWIPSER,DAA
		186.14	58.55000019	VEGELEEMER,A
930	107437.2	111.31	58.27999711	MISEFTWPNHD
		107.84	57.20000267	DVIDKQHTEQE
313	33998.1	6.04	10.54000035	GHLENNPALEK,
		10.23	24.27999973	GHLENNPALEK,
525	57169.1	21.83	26.66999996	YYVTIIDAPGHR,
		10.73	16.18999988	YYVTIIDAPGHR,

178	20291.3	4	12.91999966	YDGIILPGK,VLE(
		1.83	12.91999966	YDGIILPGK,VLE(
451	50164.3	2	25.49999952	EIIDLVLDR,SIQF
		10.12	16.62999988	EIIDLVLDR,VGIN
188	21521.2	6.03	19.67999935	ILTFDQLALDSPK
		6.94	25.52999854	GCGTVLLSGPR,
873	92991.4	4.02	4.695999995	ENAALLTANGLH
		4.35	3.206999972	MASEEEEVPLVV
661	73097.6	18.45	15.57999998	HVINFDLPSDIEE
		6.16	5.446000025	SPILVATAVAAR,
262	28714.7	4.06	8.015000075	EQGVLSFWR
		2.57	3.435000032	EQGVLSFWR
257	28024.5	3.14	10.11999995	DIIHDPGR,IDKPI
		2.69	7.393000275	AVVGVVAGGGR
1078	124922.7	85.31	43.41000021	CEEAVTTIAAYW
		77.95	40.81999958	CEEAVTTIAAYW
154	17171	2	44.15999949	TLYTSSPGGVYA
		2	44.15999949	TLYTSSPGGVYA
262	29466.3	6.63	8.015000075	LRECLPLIIFLR,L
		5.53	8.015000075	ECLPLIIFLR,LSN
524	58860.7	2	2.480999939	DFITGEVISALGF
		2.28	2.480999939	DFITGEVISALGF
2143	242910.5	2.12	0.653300015	AKLEEAILGSIGA
		4.57	1.539999992	ESILGGVLK,TILT
525	59796.8	4	3.618999943	TENELLQMFYR,
		1.31	2.095000073	TENELLQMFYR
679	73707	34.04	33.1400007	VINEPTAAALAY(
		29.98	31.07999861	RYDDPEVQK,DA
4278	480358.5	14.45	2.594999969	LSYTQLLR,QTNI
		8.25	1.331999991	RDETSGLFLLPL
588	62716.7	3.4	3.911999986	LPAHTVQDVK,Q
		2.09	2.040999942	CLSAIGTHPLAK
285	32992.6	5.91	37.54000068	DEEKMELQEIQL
		4.13	47.02000022	KLVIIEGDLER,TI
355	39421.9	14.7	26.75999999	NTQINGSWGSE
		12.96	24.51000065	SILVSGTILPNAQ
2018	228072.3	11.34	3.469000012	QDQSIILLGSSGS
		12.34	5.253000185	DLALGLVPGDR,
240	26332.4	4	7.917000353	EFLLIFR,LSEIDV
		7.36	17.08000004	EFLLIFR,FEEEIK
978	108447.5	6.87	5.418999866	SNAAAYLQHLCY
		5.34	3.475999832	GYELLFQPEVVF
642	73972.4	2	1.868999936	VLGELWPLFGG
		2	1.868999936	VLGELWPLFGG
747	81961.3	10.61	11.77999973	HVAAGTQQPYT
		4.78	4.552000016	NEGTATYAAAVL
1024	114699.9	8.4	5.273000151	LLQAVENGDAEK
		5.35	4.29700017	ESVFFAEPPFK,L
1678	191885.1	4.04	1.25099998	NLQNLLILTAIK,L
		6	2.503000014	NLQNLLILTAIK,V
1291	146666.1	82.21	40.81999958	REEESSDLLPSD

		54.51	32.37999976	TLNLTSMATK,AS
525	58222.8	14	20.19000053	HVFGQAVK,VGI
		7.55	10.85999981	CDLISIPK,VTWD
1745	202970.9	13.24	5.330000073	NCLNNFDLSEYF
		2.08	3.496000171	ETLEPLSQAAWI
214	23441.8	16.01	41.11999869	ILYSQCGDVMR,
		10.78	41.11999869	ILYSQCGDVMR,
220	25536.5	4.14	12.26999983	NCIVLIDSTPYR,I
		5.36	16.35999978	LDVGNFSWGSE
1029	115205.7	4.29	5.150999874	ITTGSSSAGTQS
		3.47	3.303999826	LAYVAPTIPR,SG
1052	116771.4	19.31	11.7899999	LQILNLGAK,LLD
		16.29	9.886000305	YAEEQQDLALLS
403	46051.6	6.17	12.40999997	SINPLGGFVHYG
		5.16	9.181000292	HGSLGFLPR,TIF
612	70349.8	9.33	11.11000031	NIYVLQELDNPG
		7.33	8.332999796	DTGIFLDLMHLK
1952	222375.8	100.28	42.32000113	HLRDQADFSVLH
		96.89	40.7799989	AELEALLSSKDD
287	32713.6	8.87	22.30000049	IPEYLTDTYFK,F
		4.33	11.15000024	QLGSGLLLVTGF
859	95451.4	4.16	2.910000086	TFCQLILDPIFK,E
		4.05	2.910000086	EGIPALDNFLDK
204	24122.8	2	5.882000178	FFEVILIDPFHK
		3.08	12.74999976	FFEVILIDPFHK,V
573	64406.8	15.13	17.10000038	ITVLEALR,YVLKI
		8	10.64999998	TAGTLFGEGFR,
886	101428.1	22.18	29.1200012	ILAGDKNYITVDE
		15.9	29.57000136	ICDQWDNLGALT
444	49670.5	25.38	37.83999979	MAVTFIGNSTAIC
		15.56	26.3500005	ISVYYNEATGGK
241	26919.2	18.17	31.94999993	SKQEALKNDLVE
		19.34	37.76000142	SKQEALKNDLVE
906	100086.4	10.89	9.713000059	LIEVANLACSISN
		3.55	2.979999967	QDLLAYLQR,LLE
724	83252.5	11.48	10.22000015	EQVANSAFVER,
		5.04	6.492000073	GVVDSEDLPLNI
161	18677.9	2	9.31700021	VYNVTQHAVGIIV
		1.4	9.31700021	VYNVTQHAVGIIV
394	44917.7	12.44	18.26999933	HLWDYTFGPEK
		18.18	39.0899986	DLMVGDEASELI
574	63210.1	8.12	11.50000021	EVPAGQYPFIVT
		4.75	5.575000122	VILPSFLVSGGD
511	57229.1	13.54	14.2900005	TASTFEDVAVVS
		14.31	18.40000004	SAVGFEYQGK,T
403	45939.1	25.47	45.41000128	YSYVCPDLVK,L
		20.27	41.94000065	AEPEDHYFLLTE
643	70400	2.01	9.019999951	LYQGAGGPGAG
		2.9	10.89000031	LYQGAGGPGAG
472	52910	36.21	42.57999957	DVDEAYMNKVE
		21.26	38.56000006	SNIDNMFESYINS

1200	135634.6	6.22	3.832999989	APEADEQELAR,
		8.2	5.917000026	DLANDIMTLMSH
967	106798.6	5.53	5.17099984	LLSIQQQLTCR,L
		6.32	5.067000166	ETIFELEDQVEQ
145	16394.9	6.66	40.68999887	VFDKDGNGYISA
		2	11.72000021	EAFSLFDKDGD(
200	22736	7.94	20.49999982	LIGNMALLPIR,N
		2.04	5.49999997	LIGNMALLPIR
751	83897.9	3.2	3.461999819	LPEELAELPLIR,(
		2	1.597999968	LPEELAELPLIR
616	68115.6	14.94	18.01999956	VVNATLWGDDA
		6.66	9.252999723	VVPIASLTPYQSI
204	22876.2	8.24	19.1200003	RVNQAIWLLCTC
		4	13.72999996	TIAECLADELINA
342	38817.3	9.65	18.70999932	RFPGYDSESK,G
		10.36	18.70999932	DIICQIAYAR,VGI
226	21694.6	21.72	45.12999952	GCLETAEEAKR,
		18.74	43.36000085	MDDSVGCLETAI
651	71431.3	25.85	22.89000005	DAGTIAGLNVLR
		20.47	24.58000034	DAGTIAGLNVLR
1930	220832.5	396.83	63.1099999	REQEVNILKK,Q\
		355.96	60.47000289	NWQWWR,ELED
171	19691.9	18	63.16000223	NAFACFDEEATC
		12.02	51.45999789	DGFIDKEDLHDM
294	33962.7	62.62	57.13999867	AELSEGQVR,KA
		58.32	61.55999899	ALMAAEDKYSQI
435	49216.2	2.01	3.448000178	WAGGGFLSTVG
		1.52	3.448000178	FGNAMLYGYQV
733	83008.1	10.01	7.639999688	CFSVLGFCR,HIE
		6.62	6.276000291	TLFPLTEVIK,TNI
299	31852.9	5.88	11.71000004	AEDKEWLPVTK,
		5.39	11.71000004	AEDKEWLPVTK,
160	18038.8	4.08	13.12000006	GPLQSVQVFGR
		2	6.25	LLEPVLLLGK
1067	121398	69.43	34.40000117	TLFATEDALELR
		64.54	34.29999948	YMDVQFDFK,LL
676	72147.9	2.02	1.627000049	AFITNIPFDVK
		2.36	1.627000049	AFITNIPFDVK
656	72554.7	9.21	16.31000042	NQLTSNPENTVF
		6.1	15.70000052	DNHLLGTFDLTG
933	105626.3	7.56	7.073999941	STESSLTPDLLN
		7.15	8.466999978	STESSLTPDLLN
534	57305.5	2.79	20.40999979	VDALTDEINFLR,
		2	16.67000055	VDALTDEINFLR
384	43718.3	2.06	2.343999967	SPLLEIVER
		11.31	22.66000062	SPLLEIVER,YLL
203	23411.9	2.72	9.359999746	YQAVTATLEEK,I
		1.43	5.418999866	YQAVTATLEEK
633	69160	9.83	11.06000021	SPSDSSTASTPV
		11.95	16.26999974	KSESEVEEAAAI
269	30252.6	5.98	13.38	LKVPPAINQFTQ

		5.68	15.98999947	LKVPPAINQFTQ
877	101401.7	50.69	40.7099992	QFASQANIVGPV
		47.43	39.21999931	NVNVQNFHISWI
435	49137.6	17.7	26.89999938	YAICSALAASALF
		8.83	22.06999958	YAICSALAASALF
2193	244870	8.34	2.37099994	EDLLQEYTEQR,
		4.84	1.64199993	YGSFEEAELSYF
1255	143051	39.34	21.51000053	RWDQGLEKPR,I
		28.69	15.53999931	VGLGLGYLELPQ
375	41736.4	2	75.73000193	DDDIAALVVDNG
		66.65	77.06999779	IWHHTFYNELR,V
286	32959.6	14.27	44.40999925	FITHAPPGEFNE
		21.89	63.63999844	TIDGQQTIIACIES

Peptide	Unique Peptide	Unique Spectrum	Spectrum	Unique Spectrum.1
1	1	1.1.1.1332.2	1	1
8	2	1.1.1.1640.13,1.1	13	4
1	1	1.1.1.1000.16	1	1
2	2	1.1.1.1698.3,1.1.:	7	7
1	1	1.1.1.1672.4,1.1.:	2	2
1	1	1.1.1.1495.25	1	1
3	1	1.1.1.1403.3	3	1
1	1	1.1.1.1789.3	1	1
1	1	1.1.1.844.30	1	1
2	2	1.1.1.1498.13,1.1	2	2
1	1	1.1.1.1233.2	1	1
12	12	1.1.1.1414.28,1.1	14	12
1	1	1.1.1.1280.6	1	1
1	1	1.1.1.941.22	1	1
1	1	1.1.1.1704.9	1	1
1	1	1.1.1.1096.34	1	1
5	4	1.1.1.1562.9,1.1.:	6	5
4	4	1.1.1.957.33,1.1.:	5	5
3	3	1.1.1.1466.5,1.1.:	3	3
7	7	1.1.1.1244.20,1.1	9	9
1	1	1.1.1.1324.17	1	1
2	2	1.1.1.1050.23,1.1	3	3
1	1	1.1.1.1568.11	1	1
3	3	1.1.1.678.3,1.1.1.	5	5
1	1	1.1.1.944.19,1.1.:	2	2
1	1	1.1.1.1512.11,1.1	2	2
1	1	1.1.1.1505.3	1	1
8	1	1.1.1.867.2	19	1
1	1	1.1.1.823.19	1	1
1	1	1.1.1.1459.7	1	1
2	2	1.1.1.790.33,1.1.:	2	2
4	4	1.1.1.1299.3,1.1.:	4	4
3	3	1.1.1.1242.17,1.1	3	3
1	1	1.1.1.1427.9	1	1
1	1	1.1.1.1569.11	1	1
1	1	1.1.1.1399.4	1	1
1	1	1.1.1.982.18	1	1
3	3	1.1.1.1373.14,1.1	3	3
6	3	1.1.1.1226.14,1.1	23	5
18	1	1.1.1.1469.4	42	1
8	2	1.1.1.1538.16	13	1
2	2	1.1.1.873.10,1.1.:	2	2
7	4	1.1.1.1735.3,1.1.:	10	5
1	1	1.1.1.1718.2,1.1.:	2	2
2	2	1.1.1.1126.8,1.1.:	2	2
1	1	1.1.1.1023.13	1	1
1	1	1.1.1.1341.2	1	1
1	1	1.1.1.1511.2	1	1
3	1	1.1.1.1266.33	3	1

1	1	1.1.1.1713.10	1	1
1	1	1.1.1.912.18	1	1
5	5	1.1.1.797.16,1.1.1	6	6
2	1	1.1.1.1375.15	3	1
1	1	1.1.1.1396.10	1	1
3	3	1.1.1.860.29,1.1.1	3	3
34	2	1.1.1.1798.6,1.1.1	362	25
1	1	1.1.1.1454.8	1	1
1	1	1.1.1.1172.3	1	1
3	3	1.1.1.1167.2,1.1.1	3	3
2	1	1.1.1.1280.11	2	1
1	1	1.1.1.1364.25	1	1
1	1	1.1.1.1422.6	1	1
1	1	1.1.1.1422.5	1	1
1	1	1.1.1.1551.10,1.1	2	2
1	1	1.1.1.1304.2	1	1
3	3	1.1.1.1305.4,1.1.1	4	4
6	6	1.1.1.985.31,1.1.1	8	8
2	2	1.1.1.1136.13,1.1	2	2
3	3	1.1.1.1217.32,1.1	3	3
15	12	1.1.1.1809.8,1.1.1	17	14
4	4	1.1.1.1247.21,1.1	7	7
1	1	1.1.1.1554.9,1.1.1	2	2
1	1	1.1.1.1296.24,1.1	2	2
3	3	1.1.1.1779.5,1.1.1	3	3
1	1	1.1.1.1176.22	1	1
9	9	1.1.1.1155.2,1.1.1	13	13
2	2	1.1.1.1124.10,1.1	3	3
1	1	1.1.1.1705.4,1.1.1	2	2
1	1	1.1.1.1475.10	1	1
2	2	1.1.1.1539.20,1.1	2	2
5	5	1.1.1.1086.10,1.1	5	5
2	2	1.1.1.1345.29,1.1	3	3
1	1	1.1.1.1531.2	1	1
1	1	1.1.1.1796.4	1	1
2	1	1.1.1.1598.16	2	1
1	1	1.1.1.930.23	1	1
11	5	1.1.1.1668.11,1.1	13	7
2	2	1.1.1.1451.7,1.1.1	3	3
2	2	1.1.1.1782.4,1.1.1	11	3
2	2	1.1.1.1301.2,1.1.1	3	3
1	1	1.1.1.1710.3	1	1
4	4	1.1.1.887.13,1.1.1	5	5
9	8	1.1.1.1253.8,1.1.1	10	9
1	1	1.1.1.1683.9	1	1
7	1	1.1.1.858.4	7	1
1	1	1.1.1.876.15	1	1
1	1	1.1.1.1402.14	1	1
1	1		1	0
1	1	1.1.1.1765.3	1	1
7	1	1.1.1.1649.12	12	1

11	10	1.1.1.1155.5,1.1.1	14	12
1	1	1.1.1.1207.21	1	1
2	1	1.1.1.1608.8	2	1
1	1	1.1.1.1136.3	1	1
1	1	1.1.1.1141.3,1.1.1	2	2
5	5	1.1.1.1599.5,1.1.1	6	6
4	4	1.1.1.1706.4,1.1.1	4	4
5	4	1.1.1.675.3,1.1.1.	5	4
6	5	1.1.1.1408.9,1.1.1	7	5
2	2	1.1.1.953.31,1.1.1	2	2
3	3	1.1.1.959.33,1.1.1	3	3
3	3	1.1.1.1574.15,1.1	3	2
1	1	1.1.1.1507.6	1	1
6	6	1.1.1.1084.27,1.1	9	9
11	11	1.1.1.869.32,1.1.1	36	28
46	2	1.1.1.1222.26,1.1	128	4
42	2	1.1.1.1233.25,1.1	131	3
2	2	1.1.1.1577.3,1.1.1	4	4
5	4	1.1.1.1605.5,1.1.1	5	4
3	3	1.1.1.1316.8,1.1.1	5	5
1	1	1.1.1.1423.2,1.1.1	2	2
5	4	1.1.1.1496.7,1.1.1	6	5
11	9	1.1.1.1464.7,1.1.1	18	11
6	6	1.1.1.895.17,1.1.1	11	11
6	6	1.1.1.1258.17,1.1	12	12
3	3	1.1.1.1534.12,1.1	4	4
3	3	1.1.1.1545.18,1.1	7	7
17	12	1.1.1.1375.4,1.1.1	24	16
13	9	1.1.1.1454.10,1.1	20	11
10	7	1.1.1.732.18,1.1.1	15	10
5	4	1.1.1.1442.24,1.1	5	4
14	7	1.1.1.1274.3,1.1.1	23	8
16	9	1.1.1.734.11,1.1.1	45	16
20	20	1.1.1.1293.5,1.1.1	37	36
20	20	1.1.1.1380.7,1.1.1	32	31
43	32	1.1.1.1488.33,1.1	212	165
35	25	1.1.1.820.2,1.1.1.	135	97
3	3	1.1.1.1654.9,1.1.1	5	5
6	6	1.1.1.1662.6,1.1.1	8	8
7	7	1.1.1.1336.7,1.1.1	11	10
9	9	1.1.1.1276.17,1.1	14	13
8	8	1.1.1.1705.12,1.1	11	11
10	10	1.1.1.908.7,1.1.1.	13	13
174	112	1.1.1.1444.35,1.1	1040	355
146	95	1.1.1.1564.4,1.1.1	970	322
62	61	1.1.1.1198.9,1.1.1	228	224
59	58	1.1.1.1148.2,1.1.1	247	240
3	3	1.1.1.1605.3,1.1.1	4	4
6	6	1.1.1.1613.6,1.1.1	9	9
12	12	1.1.1.868.3,1.1.1.	21	21
7	7	1.1.1.712.15,1.1.1	9	9

2	2	1.1.1.1121.2,1.1.	2	2
2	2	1.1.1.1231.22,1.1	2	2
8	2	1.1.1.1531.8,1.1.	14	2
5	5	1.1.1.1259.35,1.1	8	8
3	3	1.1.1.1486.19,1.1	3	3
4	4	1.1.1.1023.30,1.1	5	5
3	3	1.1.1.804.8,1.1.1.	3	3
2	2	1.1.1.829.35,1.1.	2	2
9	9	1.1.1.1516.12,1.1	10	10
3	3	1.1.1.1148.3,1.1.	3	3
2	1	1.1.1.1468.2	3	1
1	1	1.1.1.1475.2	1	1
3	3	1.1.1.716.9,1.1.1.	3	3
2	2	1.1.1.724.4,1.1.1.	2	2
46	2	1.1.1.1261.14,1.1	128	4
42	2		128	0
10	1	1.1.1.1142.13,1.1	51	7
10	1	1.1.1.963.32,1.1.	39	3
3	3	1.1.1.1800.2,1.1.	6	6
3	3	1.1.1.1810.2,1.1.	4	4
1	1	1.1.1.1679.7	1	1
1	1	1.1.1.1689.6,1.1.	2	2
1	1	1.1.1.1339.16	1	1
3	3	1.1.1.1570.13,1.1	3	3
2	2	1.1.1.1630.8,1.1.	2	2
1	1	1.1.1.1637.7	1	1
17	16	1.1.1.1612.15,1.1	55	52
16	15	1.1.1.1443.10,1.1	48	44
7	7	1.1.1.1697.8,1.1.	9	9
5	5	1.1.1.1706.7,1.1.	7	5
2	2	1.1.1.635.35,1.1.	2	2
1	1	1.1.1.864.18	1	1
17	7	1.1.1.1089.29,1.1	35	8
18	7	1.1.1.1087.23,1.1	53	5
8	8	1.1.1.1258.8,1.1.	14	14
7	7	1.1.1.1030.23,1.1	18	18
6	6	1.1.1.1319.5,1.1.	6	6
7	7	1.1.1.1504.29,1.1	8	8
2	2	1.1.1.1005.18,1.1	2	2
4	4	1.1.1.786.33,1.1.	5	5
4	4	1.1.1.1427.22,1.1	4	4
3	3	1.1.1.1816.8,1.1.	4	4
1	1	1.1.1.1773.2,1.1.	2	2
1	1	1.1.1.1782.3	1	1
6	6	1.1.1.1801.2,1.1.	6	6
3	2	1.1.1.1037.25,1.1	3	2
4	4	1.1.1.1456.7,1.1.	4	4
3	3	1.1.1.1415.15,1.1	3	3
2	2	1.1.1.1696.2,1.1.	3	3
4	4	1.1.1.1811.5,1.1.	5	5
43	43	1.1.1.762.19,1.1.	75	74

33	33	1.1.1.1153.19,1.1	54	54
9	9	1.1.1.643.4,1.1.1.	14	14
4	4	1.1.1.1157.2,1.1.1	7	7
8	5	1.1.1.981.20,1.1.1	11	5
4	1	1.1.1.1760.3,1.1.1	5	2
8	8	1.1.1.854.31,1.1.1	42	41
8	8	1.1.1.1672.8,1.1.1	22	22
2	2	1.1.1.1250.19,1.1	3	3
3	3	1.1.1.1351.11,1.1	3	3
4	4	1.1.1.646.27,1.1.1	4	4
3	3	1.1.1.686.24,1.1.1	3	3
11	11	1.1.1.1175.10,1.1	15	15
9	9	1.1.1.1578.2,1.1.1	13	13
3	3	1.1.1.1683.7,1.1.1	4	4
3	3	1.1.1.1244.6,1.1.1	3	3
5	5	1.1.1.1389.2,1.1.1	7	7
4	4	1.1.1.1401.10,1.1	4	4
83	58	1.1.1.1052.33,1.1	344	89
76	54	1.1.1.1185.14,1.1	343	92
5	5	1.1.1.1255.24,1.1	6	6
2	2	1.1.1.1668.8,1.1.1	5	5
2	2	1.1.1.1751.3,1.1.1	3	3
2	2	1.1.1.1816.7,1.1.1	3	3
1	1	1.1.1.1722.5	1	1
2	2	1.1.1.1230.22,1.1	2	2
8	8	1.1.1.1273.6,1.1.1	11	11
5	5	1.1.1.1409.10,1.1	7	7
21	11	1.1.1.1326.5,1.1.1	42	15
20	10	1.1.1.1100.3,1.1.1	46	14
13	7	1.1.1.1617.12,1.1	20	11
8	2	1.1.1.1576.16,1.1	13	2
10	10	1.1.1.1020.16,1.1	22	22
12	12	1.1.1.1189.20,1.1	17	17
6	6	1.1.1.1473.6,1.1.1	6	6
2	2	1.1.1.1662.16,1.1	2	2
6	5	1.1.1.1173.17,1.1	6	5
4	4	1.1.1.1524.20,1.1	4	4
1	1	1.1.1.1122.34,1.1	2	2
1	1	1.1.1.1128.31	1	1
6	6	1.1.1.1049.33,1.1	7	7
13	13	1.1.1.799.2,1.1.1.	26	26
4	4	1.1.1.1651.11,1.1	5	5
2	2	1.1.1.1487.25,1.1	5	5
7	7	1.1.1.957.28,1.1.1	9	9
9	9	1.1.1.1525.8,1.1.1	17	17
13	13	1.1.1.714.5,1.1.1.	26	26
11	11	1.1.1.1578.29,1.1	16	16
4	1	1.1.1.952.35	6	1
5	2	1.1.1.957.31,1.1.1	7	2
25	20	1.1.1.686.29,1.1.1	50	42
19	14	1.1.1.778.9,1.1.1.	31	25

4	4	1.1.1.1775.3,1.1.	5	5
5	5	1.1.1.1791.2,1.1.	7	7
4	4	1.1.1.1186.18,1.1	5	4
4	4	1.1.1.847.5,1.1.1.	4	4
4	4	1.1.1.1143.20,1.1	7	7
1	1	1.1.1.1257.33	1	1
4	4	1.1.1.1309.10,1.1	7	7
1	1	1.1.1.1509.3	1	1
2	2	1.1.1.1588.6,1.1.	2	2
1	1	1.1.1.1593.10	1	1
8	8	1.1.1.1199.16,1.1	9	9
4	4	1.1.1.1553.6,1.1.	5	5
4	4	1.1.1.1770.4,1.1.	12	12
2	2	1.1.1.1796.5,1.1.	3	3
5	5	1.1.1.1580.25,1.1	6	6
5	5	1.1.1.730.5,1.1.1.	10	10
25	16	1.1.1.906.17,1.1.	323	112
21	13	1.1.1.894.18,1.1.	276	82
14	11	1.1.1.1308.34,1.1	25	19
14	11	1.1.1.1021.29,1.1	21	17
232	185	1.1.1.1575.17,1.1	2912	2344
219	179	1.1.1.590.5,1.1.1.	2767	2231
9	6	1.1.1.1307.9,1.1.	38	20
8	8	1.1.1.996.16,1.1.	17	17
32	13	1.1.1.936.29,1.1.	70	27
32	21	1.1.1.1119.20,1.1	105	59
1	1	1.1.1.1801.8	1	1
1	1	1.1.1.1623.8	1	1
5	5	1.1.1.1235.5,1.1.	5	5
4	4	1.1.1.1393.2,1.1.	5	5
3	3	1.1.1.1465.5,1.1.	3	3
3	3	1.1.1.1791.3,1.1.	5	5
2	2	1.1.1.1241.7,1.1.	3	3
1	1	1.1.1.1567.3	1	1
38	38	1.1.1.629.34,1.1.	96	96
37	37	1.1.1.1694.8,1.1.	104	102
1	1	1.1.1.1486.8	1	1
1	1	1.1.1.1492.5	1	1
9	7	1.1.1.1308.19,1.1	12	7
8	6	1.1.1.1322.13,1.1	8	5
5	5	1.1.1.894.13,1.1.	6	6
6	6	1.1.1.1313.8,1.1.	6	6
10	2	1.1.1.1051.31,1.1	24	3
10	1	1.1.1.1559.4	17	1
1	1	1.1.1.1317.2	1	1
6	6	1.1.1.1231.7,1.1.	8	8
2	2	1.1.1.735.4,1.1.1.	2	2
1	1	1.1.1.947.22	1	1
5	5	1.1.1.905.32,1.1.	6	6
7	7	1.1.1.1188.11,1.1	8	8
3	3	1.1.1.937.15,1.1.	5	5

4	4	1.1.1.824.8,1.1.1.	6	6
26	16	1.1.1.1167.11,1.1	52	25
27	17	1.1.1.1384.15,1.1	56	24
11	11	1.1.1.1192.26,1.1	15	15
8	8	1.1.1.947.6,1.1.1.	10	10
4	4	1.1.1.1640.2,1.1.1	4	4
3	3	1.1.1.1528.5,1.1.1	3	3
22	22	1.1.1.1407.11,1.1	29	29
16	16	1.1.1.1420.7,1.1.1	26	25
34	2	1.1.1.1540.19,1.1	353	16
44	44	1.1.1.1422.30,1.1	675	674
8	7	1.1.1.939.35,1.1.1	13	12
12	10	1.1.1.959.31,1.1.1	35	28
